# Metabolic Potential of As-yet-uncultured Archaeal Lineages of *Candidatus* Hydrothermarchaeota Thriving in Deep-sea Metal Sulfide Deposits

**DOI:** 10.1264/jsme2.ME19021

**Published:** 2019-08-03

**Authors:** Shingo Kato, Shinsaku Nakano, Mariko Kouduka, Miho Hirai, Katsuhiko Suzuki, Takashi Itoh, Moriya Ohkuma, Yohey Suzuki

**Affiliations:** 1 Japan Collection of Microorganisms (JCM), RIKEN BioResource Research Center 3–1–1 Koyadai, Tsukuba, Ibaraki 305–0074 Japan; 2 Ore Genesis Research Unit, Project Team for Development of New-generation Research Protocol for Submarine Resources, Japan Agency for Marine-Earth Science and Technology (JAMSTEC) Yokosuka, Kanagawa, 237–0061 Japan; 3 Graduate School of Science, The University of Tokyo 7–3–1 Hongo Bunkyo-ku, Tokyo 113–0033 Japan; 4 Research and Development Center for Marine Biosciences, JAMSTEC Yokosuka, Kanagawa, 237–0061 Japan

**Keywords:** as-yet-uncultured archaea, Hydrothermarchaeota, metagenomics, deep-sea hydrothermal vents, metal sulfide deposits

## Abstract

*Candidatus* Hydrothermarchaeota, formally called Marine Benthic Group E, has often been detected in iron- and sulfur-rich marine environments, such as hydrothermal vents and cold seeps. However, their ecology and physiology remain unclear. Cultivated representatives of this group are still lacking and only several metagenome-assembled genomes (MAGs) and single-amplified genomes (SAGs) are available from two deep-sea hydrothermal areas, the Juan de Fuca Ridge (JdFR) and Guaymas Basin (GB), in the north-east Pacific. We herein report four MAGs of *Ca*. Hydrothermarchaeota recovered from hydrothermally-inactive metal sulfide deposits at the Southern Mariana Trough (SMT) in the north-west Pacific. A phylogenetic analysis indicated that the MAGs of the SMT were distinct from those of the JdFR and GB at the genus or potentially family level. *Ca*. Hydrothermarchaeota MAGs from the SMT commonly possessed putative genes for carboxydotrophic and hydrogenotrophic respiration using oxidized chemical species of sulfur as electron acceptors and also for carbon fixation, as reported previously in MAGs/SAGs from the JdFR and GB. This result strongly supports *Ca*. Hydrothermarchaeota containing anaerobic chemolithoautotrophs using carbon monoxide and/or hydrogen as electron donors. A comparative genome analysis highlighted differences in the capability of nitrogen fixation between MAGs from the SMT and the other fields, which are consistent with environmental differences in the availability of nitrogen sources for assimilation between the fields. Based on the wide distribution in various areas, abundance, and metabolic potential of *Ca*. Hydrothermarchaeota, they may play a role in the biogeochemical cycling of carbon, nitrogen, sulfur, and iron in marine environments, particularly in deep-sea hydrothermal fields.

Previous studies targeting 16S rRNA genes revealed the presence of phylogenetically diverse and as-yet-uncultured archaea in various environments, including deep-sea hydrothermal vents. Recent culture-independent single-cell genomics and metagenomics have allowed us to reconstruct the whole genomes of many as-yet-uncultured archaea, which have greatly expanded our knowledge on the ecophysiology and evolution of archaea (1, 54). In the diverse clades of as-yet-uncultured archaea, the phylogeny and ecophysiology of Marine Benthic Group E (MBGE) (60) remain poorly understood. The 16S rRNA gene sequences of MBGE have been detected as dominant archaea in sulfur- and/or iron-rich deep-sea hydrothermal environments, such as the Izu-Ogasawara arc (58), the Southern Mariana Trough (SMT) (27–29), the Okinawa Trough and Central Indian Ridge (56), the Mid-Atlantic Ridge (10), and the Juan de Fuca Ridge (JdFR) (23), implying their physiological association with iron and sulfur. In addition to deep-sea hydrothermal environments, the 16S rRNA gene sequences of MBGE have been detected in non-hydrothermal marine sediments (36, 52, 60). Thus, MBGE may contain both thermophiles and non-thermophiles, and are widely distributed in the global seafloor.

Three metagenome-assembled genomes (MAGs; JdFR-16, -17, and -18) of MBGE have recently been recovered from sub-seafloor crustal fluids in the JdFR, and the name “*Candidatus* Hydrothermarchaeota” has been proposed for MBGE (24). Furthermore, “*Ca*. Hydrothermarchaeum profundi” gen. nov., sp. nov. has been proposed to accommodate a high-quality MAG (JdFR-18) (11). Several single-amplified genomes (SAGs), all of which are closely related to the JdFR-17 MAG, has also been reported from JdFR crustal fluids (9). *Ca*. Hydrothermarchaeota appear to be an early-diverging clade in *Archaea* based on the findings of phylogenetic/phylogenomic analyses (9, 24, 54). These MAGs/SAGs harbor putative genes involved in carbon monoxide (CO) oxidation, sulfate/nitrate reduction, and carbon fixation, suggesting that members of *Ca*. Hydrothermarchaeota contribute to carbon, sulfur, and nitrogen cycling (2, 9). Additionally, two MAGs (B51_G15 and B60_G1) of *Ca*. Hydrothermarchaeota have been reported from deep-sea hydrothermal sediments in the Guaymas Basin (GB) (12). However, since only MAGs/SAGs from JdFR crustal fluids and GB hydrothermal sediments have been reported to date, the metabolic potential and ecological significance of *Ca*. Hydrothermarchaeota in other fields remain unclear.

We herein report four MAGs of *Ca*. Hydrothermarchaeota retrieved from metal sulfide deposits on and below the seafloor in SMT hydrothermal vent fields. One of the four MAGs was reconstructed from a hydrothermally inactive sulfide chimney that was newly collected in the present study. The other three MAGs were reconstructed from sub-seafloor sulfide deposits that were previously collected using a seafloor-coring system (29). The findings of analyses of 16S rRNA genes and bacterial MAGs from sub-seafloor sulfide deposits have already been reported (29, 30). A previous 16S rRNA gene analysis suggested that *Ca*. Hydrothermarchaeota predominate in the archaeal communities of sub-seafloor sulfide deposits (29). Genome information on the four MAGs of *Ca*. Hydrothermarchaeota from the SMT, in addition to previously reported MAGs/SAGs from the JdFR (9, 23) and GB (12), provide novel insights into their metabolic versatility. This versatility may explain their geographical distribution and specific adaptation to each habitat.

## Materials and Methods

### Sampling

The hydrothermally-inactive sulfide chimney (named IPdc, Fig. S1) was collected at the Pika site (40) (12°55.130′N, 143°38.972′E, a water depth of 2787 m) in the SMT using a remotely operated vehicle (ROV) *Hyper-Dolphin* (JAMSTEC, Japan) during the NT12-24 sampling cruise with the research vessel (R/V) *Natsushima* (JAMSTEC). Subsamples of the exterior and interior parts of the chimney were stored at −80°C until DNA extraction. The sample collection and preservation of sub-seafloor sulfide deposits have already been reported (29). In brief, sulfide deposits were collected from a depth of approximately 2 m below the seafloor at the Archaean site (12°56.3627′N, 143°37.9036′E, a water depth of 3,024 m), which is another hydrothermal site in the SMT, using a seafloor coring system, and were then stored at −80°C until DNA extraction.

### DNA extraction and MAG construction

Genomic DNA was extracted from subsamples (~1.0 g) of the inactive chimney by a modified method using the UltraClean Soil DNA Isolation Kit (MoBio Laboratories, Carlsbad, CA, USA) as previously described (34). Extracted DNA was used for shotgun library construction using the KAPA Hyper Prep kit for Illumina (KAPA Biosystems, Wilmington, MA, USA) (19), and the library was sequenced on an Illumina MiSeq platform (MiSeq PE300). As reported previously (30), the trimming and filtering of reads were performed using the CLC Genomics Workbench (QIAGEN Aarhus A/S), high-quality reads were assembled using SPAdes (6), the contigs produced were binned into MAGs using MetaBAT (26), and the gene prediction and annotation of MAGs were performed using Prokka (51), the Kyoto Encyclopedia of Genes and Genomes (KEGG) pathway tool (42) with the Blast-KOALA tool (25), eggNOG (21), and InterProScan (68). Protein-coding regions (CDSs) for putative c-type cytochrome (Cyc) proteins with one or more Cys-X-X-Cys-His (CXXCH) motifs and Cys-X-X-X-Cys-His (CXXXCH) motifs were extracted, and the subcellular localization of Cyc was predicted using PSORTb (67). CDSs for hydrogenase were classified using HydDB (53) and carefully checked by phylogenetic analyses as described below. Regarding sub-seafloor sulfide deposits, the MAGs of *Ca*. Hydrothermarchaeota were binned from the previously obtained assembly from two subsamples (BMS3A and BMS3B) (30) and analyzed as described above. The quality checking of MAGs was performed using CheckM (43). Average amino acid identity (AAI), which is the mean identity of all shared genes between two genomes, was calculated using Enveomics (Rodriguez-R, L.M., and K.T. Konstantinidis. 2016. The enveomics collection: A toolbox for specialized analyses of microbial genomes and metagenomes. PeerJ Preprints 4:e1900v1901.).

### Phylogenetic analysis

Phylogenetic trees of the nucleotide sequences of 16S rRNA genes and the amino acid sequences of several CDSs were constructed as previously described (30). In brief, to construct the phylogenetic tree of 16S rRNA genes, we aligned the 16S rRNA gene sequences found in the MAGs of *Ca*. Hydrothermarchaeota with the sequences of “Hydrothermarchaeota” in the Silva database release 132 (48) using SINA Aligner (47). The amino acid sequences of CDSs were aligned using MUSCLE (13). In the phylogenomic tree construction, we used a concatenated amino acid sequence of 122 conserved single-copy marker genes used in the Genome Taxonomy Database (GTDB) (44). The trimming of alignments using TrimAl (8) and maximum-likelihood (ML) tree construction using RAxML (55) were performed. Bootstrap support values were calculated with 100 replicates for all trees.

### 16S rRNA gene survey

Metadata of the16S rRNA gene sequences of *Ca*. Hydrothermarchaeota in Silva database release 132 were manually collected from original studies or public databases, such as EMBL/GenBank/DDBJ. The 16S rRNA gene sequences of *Ca*. Hydrothermarchaeota in the Sequence Read Archive (SRA) of the National Center for Biotechnology Information (NCBI), which stores raw sequence data from high-throughput sequencing platforms, such as Illumina sequencers and Roche 454 pyro-sequencers, were surveyed using IMNGS (35) at a 95% similarity threshold. The metadata of each SRA sequence, including the 16S rRNA gene sequences of *Ca*. Hydrothermarchaeota, were manually collected as described above. Location information (latitude and longitude) was extracted from these metadata, and plotted on the world map using GMT version 5.4.3 (65).

### Accession number

The raw sequence data of the inactive sulfide chimney produced by metagenomic shotgun sequencing were deposited into the DNA Data Bank of Japan under the BioProject accession number PRJDB6687 and the DDBJ SRA accession number DRA006452. The nucleotide sequences of the MAGs of BMS3Abin16, BMS3Bbin15, BMS3Bbin16, and IPdc08 were deposited into DDBJ under accession numbers BDTI01000001–BDTI01000175, BDTY01000001–BDTY01000123, BDTZ01000001–BDTZ01000197, and BHXL01000001–BHXL01000188, respectively.

## Results and Discussion

### Reconstruction of *Ca*. Hydrothermarchaeota MAGs

To obtain the MAGs of *Ca*. Hydrothermarchaeota, we performed metagenomic shotgun-sequencing of the DNAs extracted from the exterior and interior of the hydrothermally inactive sulfide chimney IPdc. After the filtering and trimming of reads, 4,155,711 and 4,311,295 high-quality reads with 274.2 and 264.7 bases on average were obtained from the exterior and interior parts, respectively. All high-quality reads were co-assembled, resulting in 37,534 contigs of 1,000 bp or longer. We obtained a MAG (IPdc08) of *Ca*. Hydrothermarchaeota by genome binning. We previously obtained high-quality reads from two sub-seafloor massive sulfide deposits (named BMS3A and BMS3B) and generated assemblies (30). In the present study, we performed genome binning from the assemblies, resulting in one MAG (BMS3Abin16) and two MAGs (BMS3Bbin15 and BMS3Bbin16) of *Ca*. Hydrothermarchaeota from BMS3A and BMS3B, respectively. In addition to the above four MAGs, we included the five MAGs of MBGE previously recovered from the JdFR sub-seafloor crustal fluids (24) and GB hydrothermal sediments (12) in our analyses. Thus, nine MAGs of MBGE were analyzed in the present study (Table 1). Although the quality checking of MAGs indicated that the two JdFR MAGs (JdFR-16 and -17) showed high contamination values (more than 10%) and low genome completeness (<55%), SAGs from the same sampling field of the JdFR (9) complemented the metabolic potential of the low-quality MAGs. The other MAGs showed 79–98% genome completeness and contamination values of less than 2%.

### Phylogeny

**Fig. 1:**
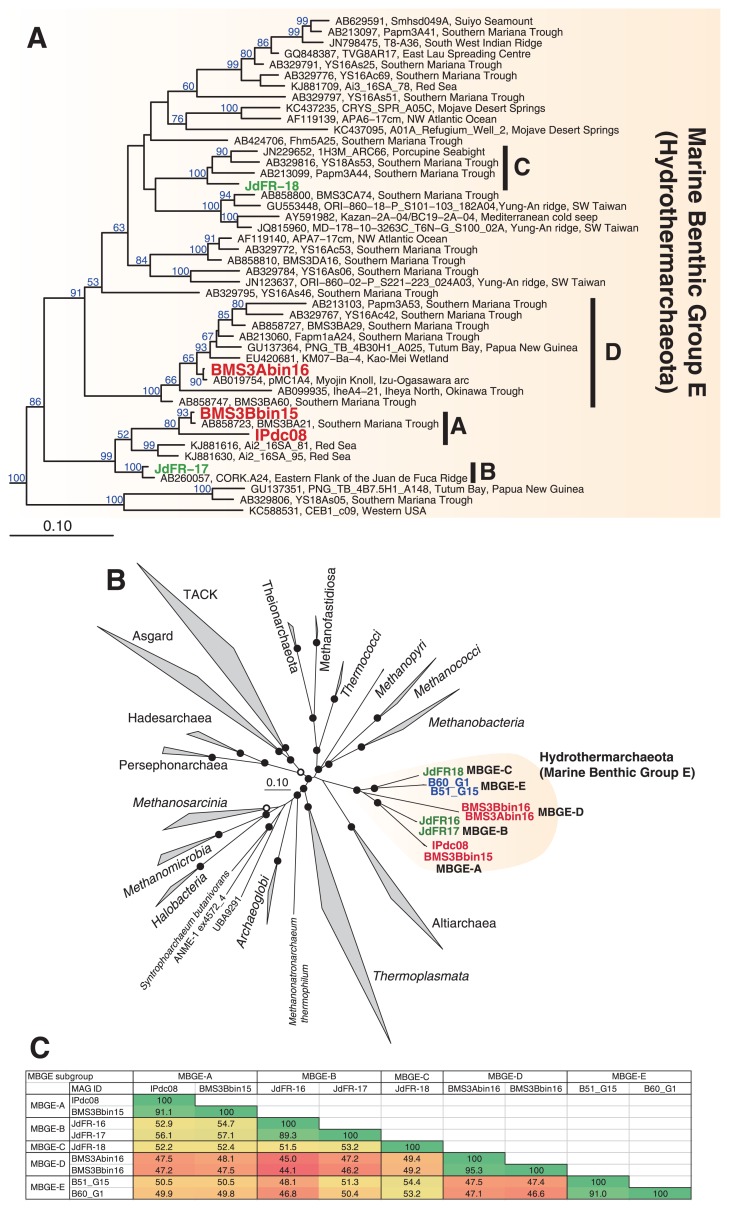
Phylogeny of MAGs of *Ca*. Hydrothermarchaeota. (A) Phylogenetic tree for the 16S rRNA gene sequences of the MAGs of *Ca*. Hydrothermarchaeota. The ID of the SMT and JdFR MAGs are in red and green, respectively. Subgroups A to D are indicated. The scale bar represents 0.1 nucleotide substitutions per sequence position. Bootstrap values (>50% of 100 replicates) are indicated. The sequences of *Ca*. Methanofastidiosa and *Ca*. Theionarchaea were used as an outgroup (not shown). (B) Phylogenomic tree for MAGs. The unrooted maximum-likelihood tree was constructed from a concatenated alignment of 122 conserved single-copy marker proteins. The ID of the SMT, JdFR, and GB MAGs are in red, green, and blue, respectively. The taxonomic names were referred to as in previous studies (1, 44). Subgroups A to E are indicated. The scale bar represents 0.1 amino acid substitutions per sequence position. Open and filled circles at branching points indicate 50–75% and >75% of bootstrap values of 100 replicates, respectively. The fast-evolving lineages of DPANN (such as Diapherotrites and Nanoarchaeota) were excluded in the tree construction to avoid long-branch attraction. (C) Average amino acid identities (%) among the MAGs of *Ca*. Hydrothermarchaeota.

Five out of the nine MAGs contained 16S rRNA gene-coding regions (Table 1). A phylogenetic analysis of 16S rRNA genes showed that MAGs were affiliated in *Ca*. Hydrothermarchaeota (Fig. 1A), which was consistent with previous findings (24). No 16S rRNA genes were found in the MAGs of BMS3Bbin16, JdFR-16, B51_G15, or B60_G1. A phylogenomic tree (Fig. 1B) indicated that all nine MAGs were clustered in a clade (*i.e*., *Ca*. Hydrothermarchaeota), and confirmed that the clade was not classified into any of the other previously reported clades, such as *Ca*. Altiarchaea (or Altiarchaeales) (46), *Ca*. Hadesarchaeota (or Hadesarchaea) (5), *Ca*. Persephonarchaea (1), *Ca*. Theionarchaeota (37), or *Ca*. Methanofastidiosa (41). Consistent with previous findings (11, 24, 44), the present results strongly supported *Ca*. Hydrothermarchaeota being a phylum- or class-level clade.

Based on phylogenetic trees (Fig. 1A and B), we separated MAGs into five subgroups: MBGE-A (BMS3Bbin15 and IPdc08), MBGE-B (JdFR-16 and JdFR-17), MBGE-C (JdFR-18), MBGE-D (BMS3Abin16 and BMS3Bbin16), and MBGE-E (B51_G15 and B60_G1). MBGE-A and -D consisted of MAGs from SMT sulfide deposits. In contrast, MBGE-B and -C consisted of MAGs from JdFR sub-seafloor crustal fluids. Based on a previous phylogenetic analysis (9), all JdFR SAGs were classified as MBGE-B. MBGE-E consisted of MAGs from GB hydrothermal sediments. AAIs among the five subgroups were <60% (Fig. 1C). Based on previous finding showing that 45%–65% of AAI may be used as the threshold for the same family (33), each subgroup may represent a genus- or potentially family-level clade.

### Metabolic potential

**Fig. 2:**
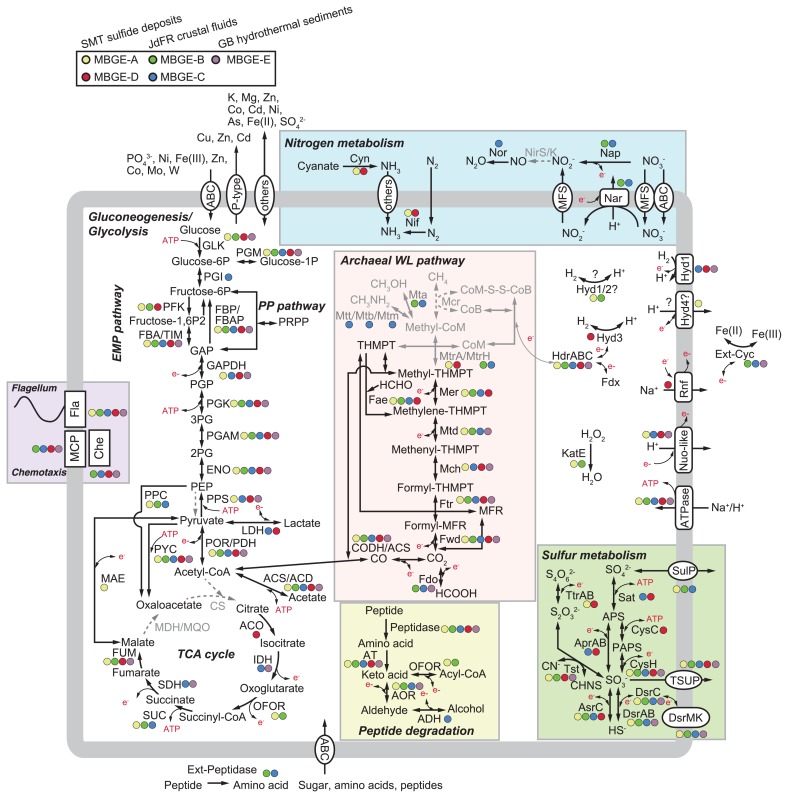
Metabolic potential of *Ca*. Hydrothermarchaeota. An overview of central metabolism and membrane transporters in *Ca*. Hydrothermarchaeota. A list of CDSs used for the metabolism reconstruction is shown in Table S1. Hyd1 to 4 indicate group 1 to 4 [NiFe]-hydrogenases. Colored circles (yellow, green, blue, red, and purple) indicate CDSs encoded in the MAGs of MBGE-A, -B, -C, -D, and -E, respectively. Pathways with CDSs found in no MAGs and with CDSs only for some of the subunits of an enzyme complex were colored in gray.

The reconstruction of metabolism suggests that *Ca*. Hydrothermarchaeota contains metabolically versatile archaea, including the capacity for hydrogenotrophy, organotrophy, sulfur/iron/nitrate reduction, autotrophy, heterotrophy, and diazotrophy. In several members, some of these capacities may overlap. The deduced metabolism and solute transport across the membrane of *Ca*. Hydrothermarchaeota from annotatable CDSs (Table S1) encoded in MAGs are summarized in Fig. 2. It is important to note that CDSs for sulfide:quinone reductase (Sqr), nitrous oxide reductase (Nos), and cytochrome c oxidase (Cox), which were only found in MBGE-B, potentially resulted from contamination from other microorganisms because of the low quality of MAGs (Table 1). Therefore, we excluded these CDSs from the discussion of their metabolic potential. Previous studies (2, 9, 12) already showed that JdFR and GB MAGs encoded CDSs for the archaeal Wood-Ljungdahl (WL) pathway for carbon fixation and CO oxidation, the sulfate adenylyltransferase (Sat)–adenosine phospho-sulfate reductase (Apr)–dissimilatory sulfite reductase (Dsr) system for dissimilatory sulfate reduction or sulfide oxidation, and group 1 [NiFe]-hydrogenase representing membrane-bound H_2_-uptake hydrogenase (15, 61). The present study showed phylogenetically distinct hydrothermarchaeotic MAGs obtained from a different environment (*i.e*., metal sulfide deposits), and greatly expands our knowledge on the metabolic potential of *Ca*. Hydrothermarchaeota as described below.

### Electron donors

Some of the SMT MAGs of MBGE-A and -D encoded CDSs of a (nearly) complete set for the archaeal WL pathway and the CO dehydrogenase catalytic subunit (*cooS*), as reported for the MAGs and SAGs of MBGE-B (9). This finding supports *Ca*. Hydrothermarchaeota containing CO oxidizers as an electron donor as previously reported (9). Furthermore, the archaeal WL pathway may be involved in acetogenesis for energy acquisition. CDSs for acetyl-CoA synthetase (ACS) or acetyl-CoA ligase (ACD) were found in the MAGs of all subgroups. CO oxidation and acetogenesis via the archaeal WL pathway have been observed in *Archaeoglobus* spp. (18). It is important to note that no CDSs for methyl coenzyme M reductase (Mcr), a key enzyme for methanogenesis, were found in MAGs.

Some members of *Ca*. Hydrothermarchaeota may also use hydrogen as an energy source, as reported previously (9). CDSs for large and small subunits of group 1j [NiFe]-hydrogenase have been found in the GB MAGs of MBGE-E (12), and the presence of these CDSs was confirmed in our phylogenetic analysis (Fig. S2). We found CDSs (BMS3Abin16_01391 and BMS3Bbin16_01271) for the large subunit of group 1 [NiFe]-hydrogenase in the SMT MAGs of MBGE-D. We also found CDSs in the JdFR MAG of MBGE-C, the presence of which has not yet been reported. Notably, our phylogenetic analysis indicated that these CDSs for the large subunit found in MBGE-C and -D constituted a clade with several archaeal homologs (Fig. S2). This clade contained no sequences of known subgroups, *i.e*., 1a to 1k (15, 53), suggesting that this clade represents a novel subgroup of group 1 [NiFe]-hydrogenase. We also found CDSs for cytochrome b and maturation factor subunits beside those for large and small subunits in each contig, similar to the case of groups 1g and 1j reported in hydrogen-oxidizing archaea (15, 53). This result suggests that the novel subgroup [NiFe]-hydrogenase is functional in hydrogen oxidation.

Our phylogenetic analysis of the large subunit of [NiFe]-hydrogenase showed that the CDSs of [NiFe]-hydrogenase reported previously in the JdFR MAGs of MBGE-B and those in one of the SMT MAG of MBGE-A may be affiliated with another novel subgroup that is located between the clades of groups 1 and 2 (Fig. S2). However, according to the classification by the HydDB Classify tool, they were classified as group 2a, the hydrogenases of which are involved in recycling H_2_ produced by cellular processes, such as fermentation and the activity of nitrogenase. We also found CDSs for several maturation factor subunits beside those for large and small subunits in each contig. Although speculative, the hydrogenases of the novel subgroup may be involved in hydrogen oxidation coupled with sulfate/nitrate reduction, or in hydrogen evolution as a sink for the electrons produced during CO oxidation, as previously reported (9).

CDSs for group 3 [NiFe]-hydrogenase were only found in one of the SMT MAGs of MBGE-D. The phylogenetic analysis and HydDB classification indicated that CDSs were classified as group 3c (Fig. S2), the hydrogenases of which are involved in electron bifurcation from H_2_ to heterodisulfide and ferredoxin. However, no homologs of the FeS and polyferredoxin subunits, which are involved in electron transfer, for group 3c [NiFe]-hydrogenase were found. Therefore, it currently remains unclear whether the CDSs found in the SMT MAG are involved in electron bifurcation.

CDSs for group 4 [NiFe]-hydrogenase were only found in the SMT MAGs of MBGE-A. The phylogenetic analysis indicated that CDSs were closely related to those of group 4f (Fig. S2), the hydrogenases of which are considered to be involved in hydrogen evolution coupled with formate oxidation. Notably, these CDSs did not clearly belong to group 4f, but rather constituted a distinct clade with some bacterial and archaeal homologs. Several CDSs for transmembrane subunits were found beside the large and small subunits in each contig, indicating that the protein complex is located in the cell membrane. The functional role of the protein complex in this novel clade remains unclear.

In addition to their capability for CO and H_2_ oxidation, some members of *Ca*. Hydrothermarchaeota may use organic carbon compounds, including acetate, lactate, formate, alcohol, aldehyde, peptides, and/or amino acids, as energy and carbon sources. The MAG JdFR-18 of MBGE-C encoded CDSs for lactate dehydrogenase (LDH), alcohol dehydrogenase (ADH), aldehyde:ferredoxin oxidoreductase (AOR), and formate dehydrogenase (Fdo), whereas other MAGs encoded some or none of these CDSs. CDSs for extracellular peptidase were only found in the JdFR MAGs of MBGE-B and -C. No MAGs had the complete set of CDSs for the TCA cycle. No CDSs for malate dehydrogenase (MDH) or citrate synthase (CS) were found in MAGs.

CDSs for NADH:ubiquinone oxidoreductase (Nuo) and ferredoxin:NAD+ oxidoreductase (Rnf) were found in some of the MAGs of *Ca*. Hydrothermarchaeota. We found all CDSs for the 11-subunit type of the NADH:ubiquinone oxidoreductase (Nuo) complex (39) in one of the SMT MAGs of MBGE-A (Table S1). Furthermore, several parts of the 11 subunits were found in the MAGs/SAGs of MBGE-B, -C, -D, and -E (Table S1) (9, 12). As reported in the 11-subunit or other types of the Nuo-like complex (50), all MAGs/SAGs of *Ca*. Hydrothermarchaeota were lacking the homologs of NuoEFG subunits for the NADH dehydrogenase module of the standard Nuo complex. A phylogenetic analysis of the CDSs for NuoD (Fig. S3) indicated that those of MBGE-A, -C, and -E were closely related to some archaeal homologs and did not belong to the cluster of F_420_H_2_ dehydrogenase (Fpo) or a membrane-bound hydrogenase-like complex (Mbx). The CDS of MBGE-D was related to the Fpo cluster. A ferredoxin:methanophenazine oxidoreductase reported from *Methanothrix thermoacetophila* (also known as *Methanosaeta thermophila*) has been reported in the Fpo cluster (64). In contrast to CDSs for the Nuo-like complex, those for Rnf are only found in the SMT MAGs of MBGE-D. Overall, these CDSs for the Nuo-like complex and Rnf may be involved in Na^+^/H^+^ translocation across the membrane, and in electron transfer between co-factors, such as ferredoxin, F_420_, quinone, and NAD^+^.

### Carbon fixation

*Ca*. Hydrothermarchaeota may commonly fix inorganic carbon via the archaeal WL pathway and grow autotrophically as previously reported (9). In addition to the archaeal WL pathway, CDSs for ribulose-1,5-bisphophate carboxylase-oxygenase (RubisCO), which is a key enzyme for carbon fixation via the Calvin–Benson–Bassham (CBB) cycle, were found in the MAGs of all subgroups, except MBGE-A (Table S1). A phylogenetic analysis indicated that the CDSs of RubisCO were classified into Forms III-a, III-b, and IV (Fig. S4) based on the previous classification of RubisCO (32, 57, 66). The CDSs of Forms III-a and III-b may be involved in the reductive hexulose-phosphate (RHP) pathway (32) and adenosine 5′-monophosphate (AMP) metabolism (50), respectively. Although CO_2_ is fixed into organic compounds via the RHP pathway and AMP metabolism, it currently remains unclear whether they support autotrophic growth (32, 49). The CDSs of Form IV may be involved in other metabolic pathways, such as the methionine salvage pathway and sulfur metabolism (3, 17), but not in carbon fixation. No CDSs for key enzymes for other carbon fixation pathways (such as the reductive citric acid cycle and hydroxypropionate–hydroxybutyrate cycle) were found.

### Reduction of sulfate and other sulfur species

**Fig. 3:**
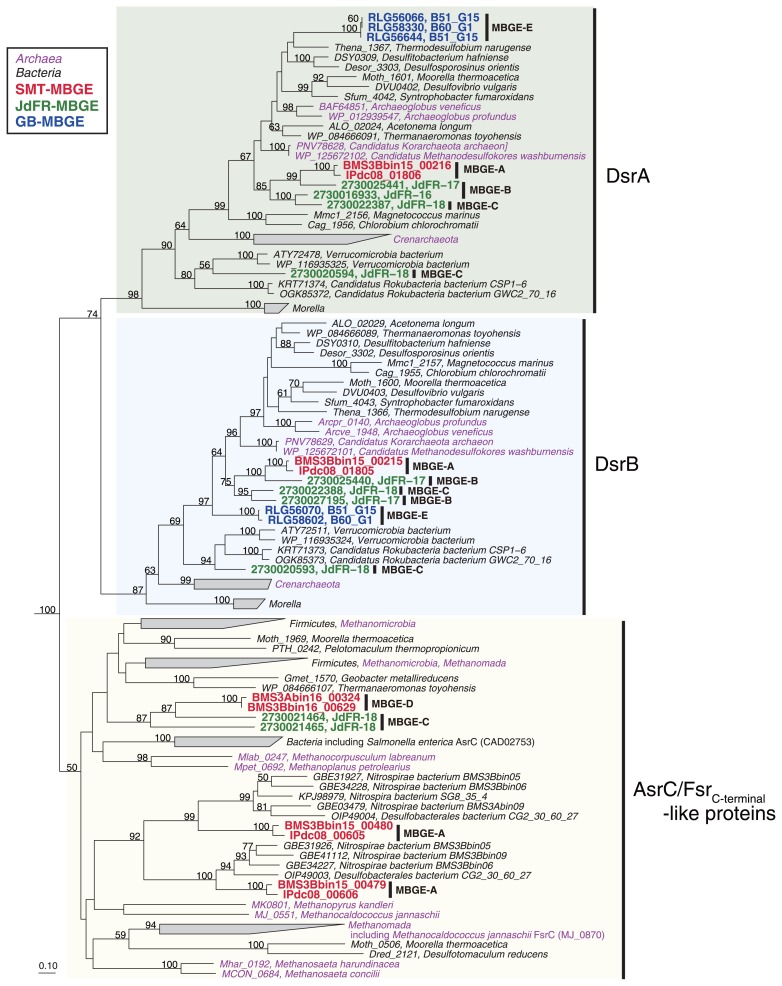
Phylogeny of CDSs related to DsrAB and AsrC. All protein sequences in the phylogenetic tree were categorized in COG2221, including DsrA, DsrB, AsrC, and the C terminus of Fsr. The ID of SMT, JdFR, and GB MAGs are in red, green, and blue, respectively. The species names colored in black and magenta indicate bacterial and archaeal species, respectively. The sequences of sulfite reductase (NCBI accession numbers, AAA23651 and CAA89154) were used as an outgroup (not shown). The scale bar represents 0.1 amino acid substitutions per sequence position. Bootstrap values (>50%) are indicated.

Some members of *Ca*. Hydrothermarchaeota may reduce sulfate and intermediate sulfur species (ISS), such as sulfite and tetrathionate, as electron acceptors. All CDSs for Sat, AprAB, and DsrABC, which are key enzymes for dissimilatory sulfate reduction, were found in the MBGE-C MAG (Fig. 2) as previously reported (2). No CDSs for DsrD and DsrEFH, which are potential marker proteins for sulfate reduction and sulfide oxidation, respectively, for the Dsr system (16), were found in the MAGs, which was similar to the genomes of sulfate-reducing crenarchaeota, such as *Caldivirga maquilingensis* and *Vulcanisaeta distributa* (2). The MAGs of MBGE-D encoded CDSs for Sat and Apr, but not CDSs for Dsr. Alternatively, these MAGs encoded CDSs for the homologs of anaerobic sulfite reductase subunit C (AsrC) (20) or the C terminus of coenzyme F_420_-dependent sulfite reductase (Fsr-C) (Fig. 3) (22). Notably, the CDSs for Sat, AprAB, and AsrC were continuously located in a contig (Table S1) as a gene cluster. This result prompted us to speculate that these CDSs are collectively involved in sulfate reduction, although such a Sat-Apr-Asr system has not been reported in any sulfate-reducing organisms to date. The cysteine residues for the siroheme-binding site (CX_5_CX_n_CX_3_C) were found in the CDSs for DsrA, DsrB, and AsrC/Fsr-C (Fig. S5). Tetrathionate may be reduced to thiosulfate by tetrathionate reductase subunits A and B (TtrAB), which were only found in the SMT MAGs of MBGE-A and -D. If cyanide is available, sulfite may be produced from thiosulfate by thiosulfate sulfurtransferase (Tst). We cannot exclude the possibility that the above reduction pathways of sulfate and ISS are used for sulfide or ISS oxidation, as previously reported for *Desulfurivibrio alkaliphilus* (59). Further analyses, such as cultivation and physiological characterization, are needed to assess the roles of these CDSs in their sulfur metabolism.

### Nitrogen fixation and nitrate reduction

The members of *Ca*. Hydrothermarchaeota in sulfide deposits in the SMT may contain diazotrophs that fix nitrogen. CDSs for nitrogenase (NifHDKBI1I2) were only found in the MAGs of MBGE-A and -D (Fig. 2). In addition, these MAGs encoded cyanate lyase (Cyn), which catalyzes the conversion from cyanate to ammonia. The ammonia produced from nitrogen and cyanate may be used for nitrogen assimilation. This is the first study to show the presence of Nif and Cyn in the genomes of *Ca*. Hydrothermarchaeota.

The members of *Ca*. Hydrothermarchaeota in the sub-seafloor crustal fluids of the JdFR may contain nitrate reducers, as previously reported (9). CDSs for dissimilatory nitrate reductase (Nar) and periplasmic nitrate reductase (Nap) were only found in the MAGs of MBGE-B and -C. Although CDSs for nitric oxide reductase (Nor) involved in the denitrification pathway were found in MAGs, no CDSs for nitrite reductase (NirS/K) were detected.

### Extracellular electron transfer

The JdFR and GB members of *Ca*. Hydrothermarchaeota may contain iron reducers or syntrophs that transfer electrons to a syntrophic partner. The MAGs of all subgroups encoded CDSs for multi-heme *c*-type cytochromes (MHCs) with heme-binding motifs (CXXCH or CXXXCH) (Table S2). Archaeal MHCs at the cell wall and extracellular space may be involved in electron transfer to extracellular solid Fe(III) oxides (7, 14, 31) or to symbiotic partners, such as sulfate reducers (38, 63). Although the presence of MHCs in the JdFR MAGs of MBGE-B and -C was previously reported (9), the number of heme-binding motifs and cellular sub-locations were unclear. Notably, ten or more (up to 67) motifs were found in the protein sequences of the MHC CDSs in the JdFR MAGs of MBGE-B and -C. The GB MAGs of MBGE-E also contained MHC CDSs with up to 10 motifs. Some of the MHC CDSs of the JdFR and GB MAGs were predicted to be located at the cell wall or extracellular space (Table S2). In contrast, MHC CDSs in the SMT MAGs of MGBE-A and -D had up to 5 motifs, and none of the CDSs were predicted to be located at the cell wall or extracellular space. Thus, the SMT members of *Ca*. Hydrothermarchaeota did not appear to contain iron reducers or syntrophs.

### Differences in metabolic potential for elemental cycling

Genomic comparisons among the SMT, JdFR, and GB members of *Ca*. Hydrothermarchaeota (Fig. 2) highlighted differences in metabolic potential for nitrogen and iron cycling. Regarding nitrogen sources for assimilation, the sub-seafloor crustal fluids of the JdFR (sampling sites, U1362A and U1362B) showed high concentrations of ammonia (~100 μM) (23). GB hydrothermal sediments also contained high concentrations of ammonia (several tens of mM) (62). Therefore, the JdFR and GB members may uptake ammonia for assimilation, and may not need to fix nitrogen. This is a reasonable explanation for the absence of the CDSs of Nif in the JdFR and GB MAGs. In contrast, ammonia (in general, tens of nM in deep-sea water) must be limited in sulfide deposits, although its actual concentrations in the porewater of metal sulfide deposits have not yet been measured. Dominant bacterial members (such as *Nitrospirae* and *Deltaproteobacteria*) in sub-seafloor sulfide deposits in the SMT encoded Nif for nitrogen fixation (30). In this environment, members of *Ca*. Hydrothermarchaeota may also need to fix nitrogen (or degrade cyanate) to ammonia for assimilation. Thus, the presence/absence of Nif in MAGs may reflect environmental differences among the habitats.

Regarding electron acceptors, difficulties are associated with linking the metabolic difference to the environmental difference. Although nitrate (generally several tens of μM in deep-sea water) may be available in SMT sub-seafloor sulfide deposits (29) and GB hydrothermal sediments (45), SMT and GB MAGs did not encode CDSs for dissimilatory nitrate reductase (*i.e*., Nar and Nap). In contrast, the JdFR MAGs encoded these CDSs despite nitrate concentrations being relatively low (<2.0 μM) in the sub-seafloor crustal fluids of the JdFR (23). Although Fe(III) oxides may be available in SMT sub-seafloor sulfide deposits (29) and the bacterial MAGs encoded CDSs for extracellular MHCs (30), the SMT MAGs of *Ca*. Hydrothermarchaeota did not encode these CDSs. In contrast, JdFR MAGs encoded extracellular MHCs with a higher number of heme-binding motifs; however, the majority of total dissolved iron (<2.4 μM) was ferrous iron in the sub-seafloor crustal fluids of the JdFR (24). Further analyses, such as the cultivation and physiological characterization of *Ca*. Hydrothermarchaeota and others, and more detailed measurements using *in situ* chemistry are needed to elucidate the relationship between the predicted metabolism of *Ca*. Hydrothermarchaeota and the surrounding environmental factors.

### Global distribution and relative abundance

**Fig. 4:**
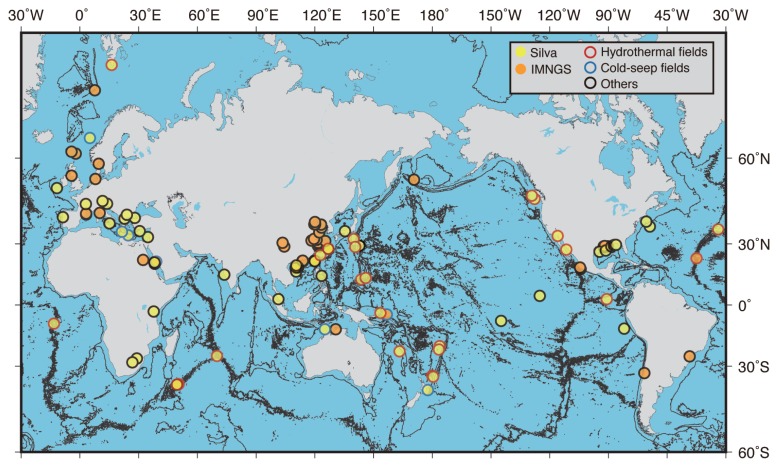
Global distribution of 16S rRNA genes of *Ca*. Hydrothermarchaeota. The locations at which the 16S rRNA genes of *Ca*. Hydrothermarchaeota were detected are plotted. Yellow and orange dots indicate the locations by the Silva and IMNGS tools used in the analysis, respectively. Locations with red, blue, and black circles indicate hydrothermal vent fields, cold-seep fields, and others, respectively. Details of locations are shown in Table S3 for Silva and Table S4 for IMNGS.

To assess the global distribution of *Ca*. Hydrothermarchaeota, we surveyed their 16S rRNA gene sequences deposited into public databases. The locations at which they were detected are mapped in Fig. 4. Metadata for the sequences typically elucidated by Sanger sequencing are summarized in Table S3, while those by next-generation sequencing, such as pyrosequencing and Illumina sequencing, are shown in Table S4. The 16S rRNA gene sequences of *Ca*. Hydrothermarchaeota have mainly been detected in marine environments, such as deep-sea and coastal sediments and seawater, and sometimes in terrestrial environments, including lakes, subsurface groundwater, and hot springs. In some deep-sea hydrothermal deposits and cold-seep sediments, they have accounted for >50% of the total number of analyzed sequences for each sample, and for >10% of the total number of prokaryotic cells estimated by quantitative PCR or direct counting by fluorescence *in situ* hybridization (Table S3 and S4). Based on the read coverage of ribosomal protein S3 genes (rps3), some of the single copy marker genes used in abundance analyses (*e.g*., [4]), *Ca*. Hydrothermarchaeota were represented in the top 20 abundant members in the SMT sulfide deposits used in the present study (Fig. S6). Based on their global distribution, abundance, and metabolic potential, as described above and reported previously (9, 12), *Ca*. Hydrothermarchaeota appear to be widely distributed in cold to hot marine environments (and sometimes in terrestrial aquifers) with various chemistries, and, in some cases, may significantly contribute to the biogeochemical cycling of carbon, nitrogen, sulfur, and potentially iron. Their metabolic versatility may allow for adaptation to diverse environments.

## Supplementary information



## Figures and Tables

**Table 1:** List of MAGs of *Ca*. Hydrothermarchaeota used in the present study

MAG ID	MBGE Subgroup^a^	Comp^b^	Cont^b^	Total length (bp)	Estimated genome size (Mbp)^c^	GC content	Number of rRNAs	Number of CDSs	Number of contigs	Contig N50 (bp)	Longest contig (bp)
BMS3Bbin15	A	97.2%	0.93%	1738368	1.79	40.1%	16S ×1, 23S ×1, 5S ×1	1951	123	20447	81722
IPdc08	A	92.1%	1.25%	1695311	1.84	39.2%	16S ×1, 5S ×1	1897	188	11496	31809
JdFR-16^d^	B	31.9%	13.6%	1353114	4.24	49.7%	Not found	1680	241	6264	45234
JdFR-17^d^	B	54.7%	28.7%	2178134	3.98	49.6%	16S ×2, 5S ×1	2717	344	7692	39644
JdFR-18^d^	C	98.1%	1.87%	2062134	2.10	39.1%	16S ×1, 23S ×1, 5S ×1	2269	22	149031	364381
BMS3Abin16	D	90.2%	1.87%	1628016	1.80	46.7%	16S ×1, 23S ×1, 5S ×1	1863	175	12106	34655
BMS3Bbin16	D	79.0%	0%	1164696	1.47	46.9%	5S ×1	1292	197	6378	25078
B51_G15^e^	E	71.2%	4.00%	985975	1.38	38.5%	5S ×1	1204	218	4521	15462
B60_G1^e^	E	72.6%	9.55%	1504894	2.07	39.9%	5S ×1	1817	398	3989	15453

a based on the phylogeny shown in Fig. 1;

b completeness (Comp) and contamination (Cont) values calculated using CheckM in this study;

c calculated based on the total length of the MAGs and genome completeness values;

d MAGs reported previously (Jungbluth *et al*., 2017);

e MAGs reported previously (Dombrowski *et al*., 2018).
