# Supplementary material for: Metabolic Potential of As-yet-uncultured Archaeal Lineages of Candidatus Hydrothermarchaeota Thriving in Deep-sea Metal Sulfide Deposits
Source: Microbes Environ. 2019 Aug 3;34(3):293–303. doi: 10.1264/jsme2.ME19021 (PMC6759336; doi:10.1264/jsme2.ME19021)
Supplement: Supplementary file 1 [file 34_293_s1.pdf]

Supplementary information

Metabolic potential of as-yet-uncultured archaeal lineages of *Candidatus* Hydrothermarchaeota thriving in deep-sea metal sulfide deposits

Shingo Kato*, Shinsaku Nakano, Mariko Kouduka, Miho Hirai, Katsuhiko Suzuki, Takashi Itoh, Moriya Ohkuma, and Yohey Suzuki

*Corresponding author
Shingo Kato, Ph.D.
Japan Collection of Microorganisms (JCM), RIKEN BioResource Research Center
3-1-1 Koyadai, Tsukuba, Ibaraki 305-0074, Japan
Tel: +81-29-846-0012, FAX: +81-29-836-9562
E-mail: skato@riken.jp

Supplementary tables

Table S1. List of CDSs used in Fig. 2

Table S2. List of CDSs for *c*-type cytochromes encoded in the MAGs of *Ca.* Hydrothermarchaeota

Table S3. List of the locations where 16S rRNA genes of *Ca*. Hydrothermarchaeota were detected and other metadata (Silva database)

Table S4. List of the locations where 16S rRNA genes of *Ca*. Hydrothermarchaeota were detected and other metadata (IMNGS)

Supplementary figures

Fig. S1. Photo of the inactive chimney used in this study. The photo was taken at the Pika site in the Southern Mariana Trough during the dive HPD#1435. The white arrow indicates the chimney sample used in this study.

Fig. S2. Phylogeny and subunit component of CDSs for hydrogenase. The phylogenetic tree for the large subunits of the groups 1 to 4 of [NiFe] hydrogenase is shown. The ID of the SMT, JdFR, and GB MAGs are in red, green, and blue, respectively. The species names colored in black and magenta indicated bacterial and archaeal species, respectively. The arrows colored in blue, green, orange, yellow, and purple, indicate CDSs for small, large, maturation factor, cytochrome *b*, and transmembrane subunits, respectively. The midpoint between the group 4 and the others was rooted. The scale bar represented 0.3 amino acid substitutions per sequence position. Bootstrap values (> 50%) were indicated.

Fig. S3. Phylogeny of CDSs for NuoD homologs. The ID of the SMT, JdFR, and GB MAGs are in red, green, and blue, respectively. The species names colored in black and magenta indicated bacterial and archaeal species, respectively. The midpoint between hydrogenases (such as EchE, EhaO, and HyfG) and the others was rooted. The scale bar represented 0.1 amino acid substitutions per sequence position. Bootstrap values (> 50%) were indicated.

Fig. S4. Phylogeny of CDSs for RubisCO. Form I to IV of RubisCO were indicated. The ID of the SMT, JdFR, and GB MAGs are in red, green, and blue, respectively. The species names colored in black and magenta indicated bacterial and archaeal species, respectively. The midpoint between Form II-Intermediate form II/III and the others was rooted. The scale bar represented 0.1 amino acid substitutions per sequence position. Bootstrap values (> 50%) were indicated.

Fig. S5. Alignment of amino acid sequences of DsrA, DsrB, and AsrC/Fsr$_{\text{C-terminal}}$. Only the regions including siroheme-[4Fe4S] binding sites are shown.

Fig. S6. Abundance of *Ca.* Hydrothermarchaeota in the metal sulfide deposits. Rank-abundance plots of the read coverages of contigs containing ribosomal protein S3 genes in (A) IPdc, (B) BMS3A and (C) BMS3B metagenomes, are shown. (B and C) Modified from Kato et al. (2018). The contigs for the MAGs of *Ca.* Hydrothermarchaeota are indicated on the bars.

Supplementary reference

Kato, S., T. Shibuya, Y. Takaki, M. Hirai, T. Nunoura, and K. Suzuki. 2018. Genome-enabled metabolic reconstruction of dominant chemosynthetic colonizers in deep-sea massive sulfide deposits. Environ Microbiol 20:862-877.

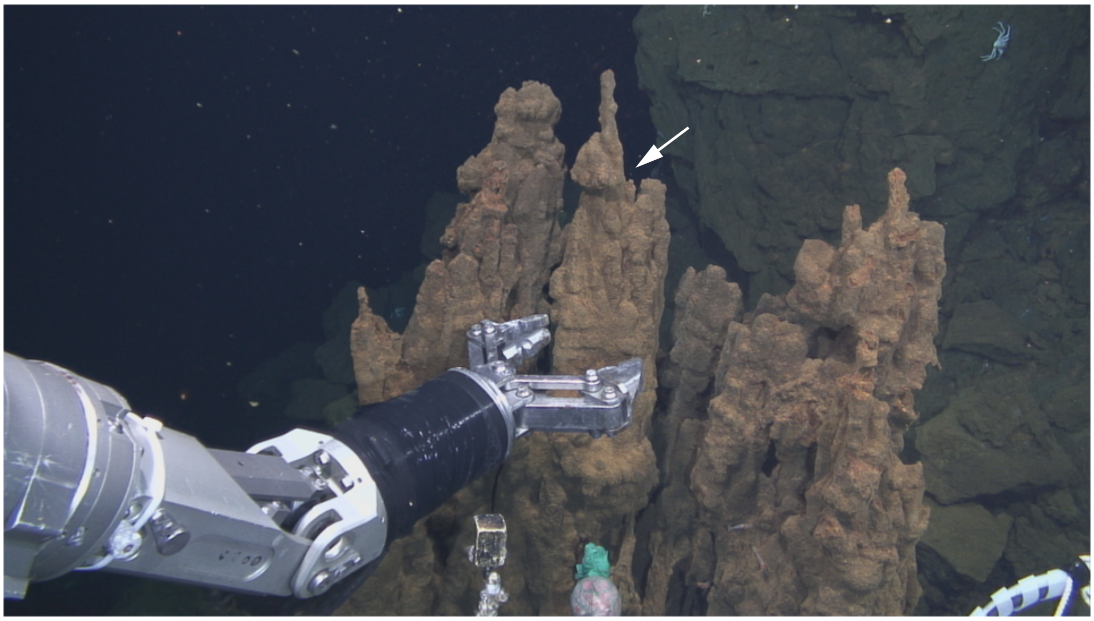

Fig. S1

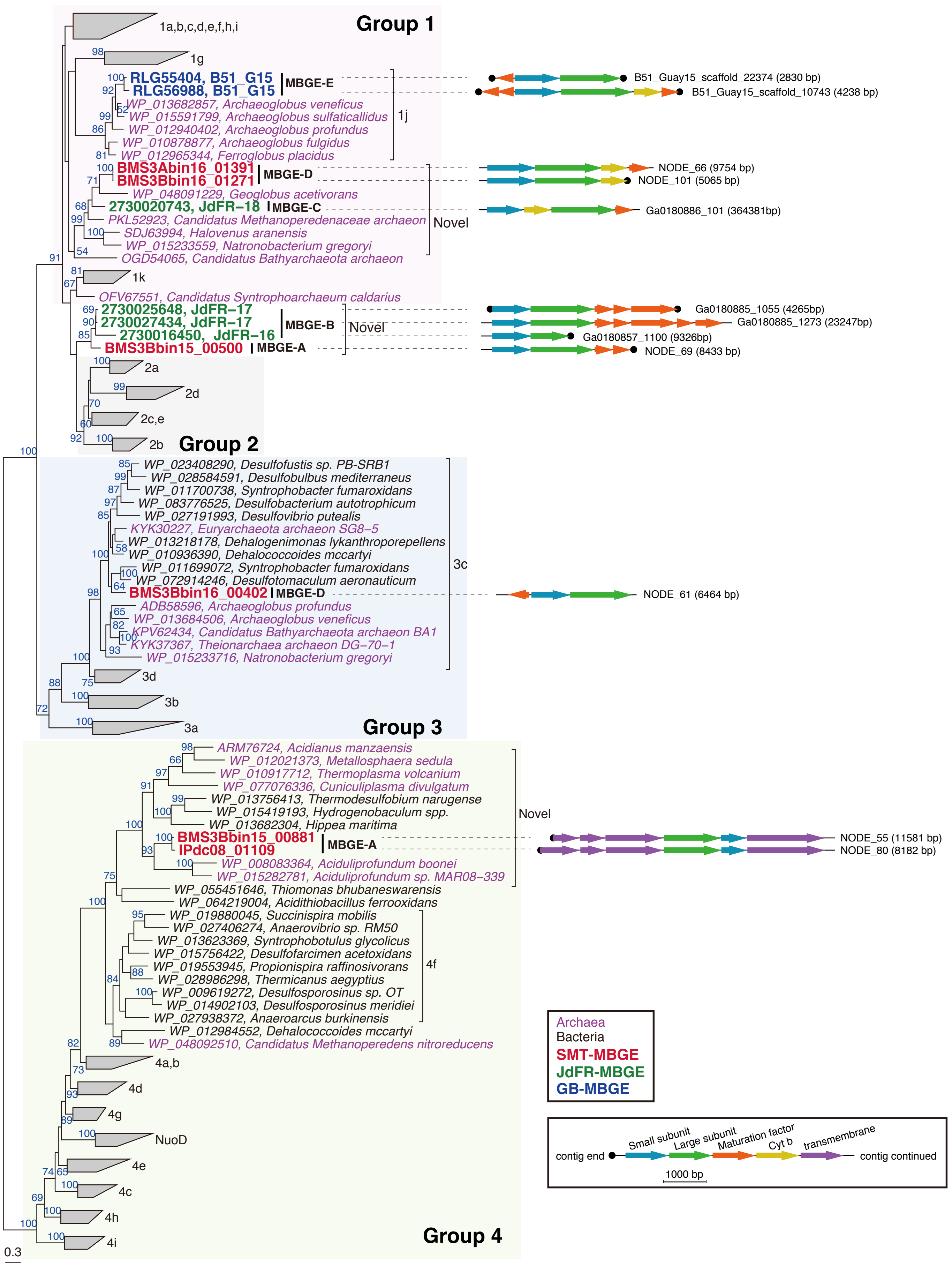
Group 1
1a,b,c,d,e,f,h,i
1g
RLG55404, B51_G15
RLG56988, B51_G15
MBGE-E
WP_013682857, Archaeoglobus veneficus
WP_015591799, Archaeoglobus sulfaticallidus
WP_012940402, Archaeoglobus profundus
WP_010878877, Archaeoglobus fulgidus
WP_012965344, Ferroglobus placidus
1j
B51_Guay15_scaffold_22374 (2830 bp)
B51_Guay15_scaffold_10743 (4238 bp)
BMS3Abin16_01391
BMS3Bbin16_01271
MBGE-D
WP_048091229, Geoglobus acetivorans
2730020743, JdFR-18
MBGE-C
PKL52923, Candidatus Methanoperedenaceae archaeon
SDJ63994, Halovenus aranensis
WP_015233559, Natronobacterium gregoryi
OGD54065, Candidatus Bathyarchaeota archaeon
Novel
NODE_66 (9754 bp)
NODE_101 (5065 bp)
Ga0180886_101 (364381bp)
1k
OFV67551, Candidatus Syntrophoarchaeum caldarius
2730025648, JdFR-17
2730027434, JdFR-17
2730016450, JdFR-16
MBGE-B
BMS3Bbin15_00500
MBGE-A
Novel
Ga0180885_1055 (4265bp)
Ga0180885_1273 (23247bp)
Ga0180857_1100 (9326bp)
NODE_69 (8433 bp)
2a
2d
2c,e
2b
Group 2
WP_023408290, Desulfofustis sp. PB-SRB1
WP_028584591, Desulfobulbus mediterraneus
WP_011700738, Syntrophobacter fumaroxidans
WP_083776525, Desulfobacterium autotrophicum
WP_027191993, Desulfovibrio putealis
KYK30227, Euryarchaeota archaeon SG8-5
WP_013218178, Dehalogenimonas lykanthroporepellens
WP_010936390, Dehalococcoides mccartyi
WP_011699072, Syntrophobacter fumaroxidans
WP_072914246, Desulfotomaculum aeronauticum
BMS3Bbin16_00402
MBGE-D
ADB58596, Archaeoglobus profundus
WP_013684506, Archaeoglobus veneficus
KPV62434, Candidatus Bathyarchaeota archaeon BA1
KYK37367, Theionarchaea archaeon DG-70-1
WP_015233716, Natronobacterium gregoryi
3c
NODE_61 (6464 bp)
3d
3b
3a
Group 3
ARM76724, Acidianus manzaensis
WP_012021373, Metallosphaera sedula
WP_010917712, Thermoplasma volcanium
WP_077076336, Cuniculiplasma divulgatum
WP_013756413, Thermodesulfobium narugense
WP_015419193, Hydrogenobaculum spp.
WP_013682304, Hippea maritima
BMS3Bbin15_00881
IPdc08_01109
MBGE-A
WP_008083364, Aciduliprofundum boonei
WP_015282781, Aciduliprofundum sp. MAR08-339
Novel
NODE_55 (11581 bp)
NODE_80 (8182 bp)
WP_055451646, Thiomonas bhubaneswarensis
WP_064219004, Acidithiobacillus ferrooxidans
WP_019880045, Succinispira mobilis
WP_027406274, Anaerovibrio sp. RM50
WP_013623369, Syntrophobotulus glycolicus
WP_015756422, Desulfofarcimen acetoxidans
WP_019553945, Propionispira raffinosivorans
WP_028986298, Thermicanus aegyptius
WP_009619272, Desulfosporosinus sp. OT
WP_014902103, Desulfosporosinus meridiei
WP_027938372, Anaeroarcus burkinensis
4f
WP_012984552, Dehalococcoides mccartyi
WP_048092510, Candidatus Methanoperedens nitroreducens
4a,b
4d
4g
NuoD
4e
4c
4h
4i
Group 4
0.3
Archaea
Bacteria
SMT-MBGE
JdFR-MBGE
GB-MBGE
contig end
Small subunit
Large subunit
Maturation factor
Cyt b
transmembrane
contig continued
1000 bp

Fig. S2

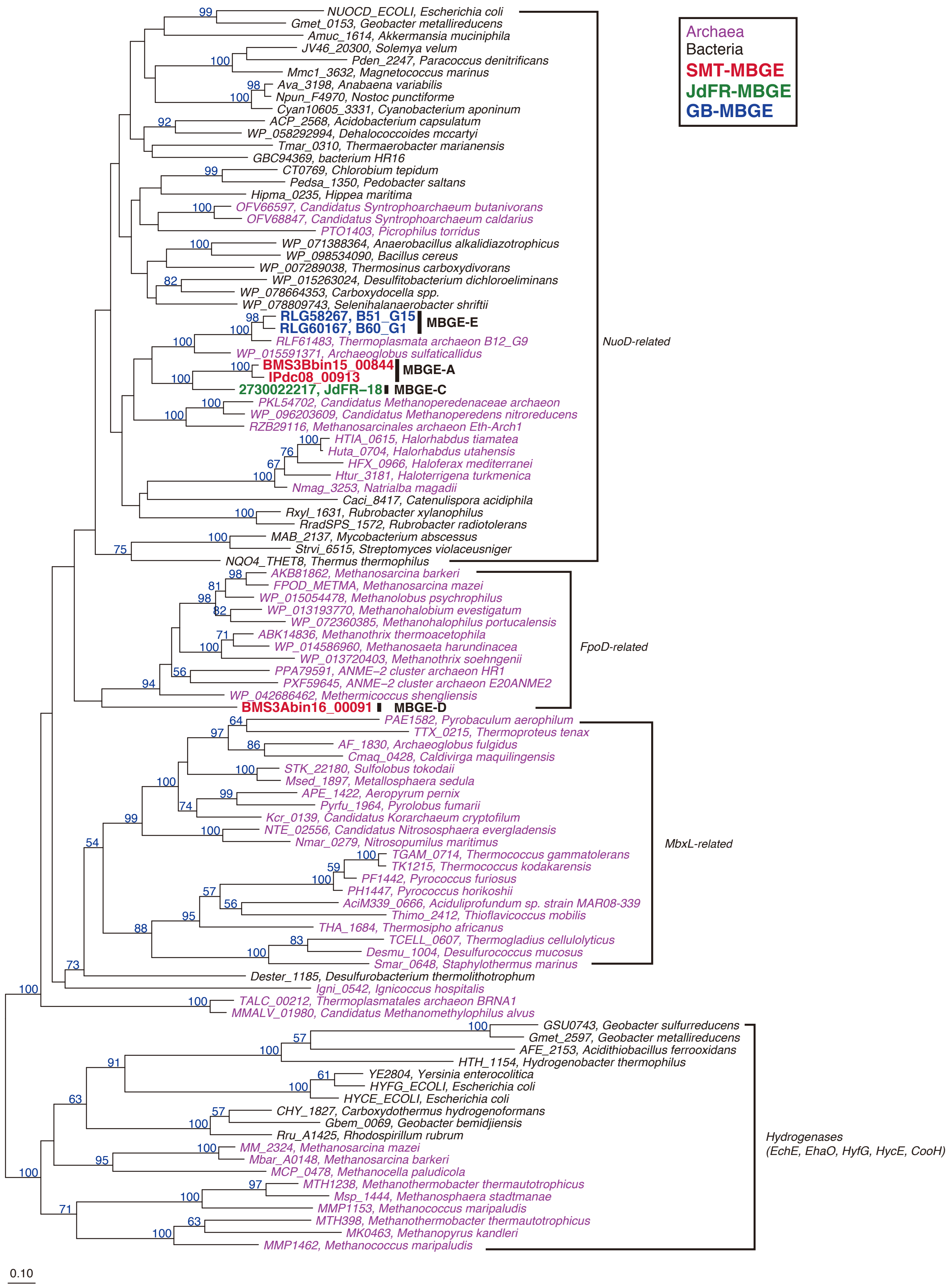

Archaea
Bacteria
SMT-MBGE
JdFR-MBGE
GB-MBGE
NUOCD_ECOLI, Escherichia coli
Gmet_0153, Geobacter metallireducens
Amuc_1614, Akkermansia muciniphila
JV46_20300, Solemya velum
Pden_2247, Paracoccus denitrificans
Mmc1_3632, Magnetococcus marinus
Ava_3198, Anabaena variabilis
Npun_F4970, Nostoc punctiforme
Cyan10605_3331, Cyanobacterium aponinum
ACP_2568, Acidobacterium capsulatum
WP_058292994, Dehalococcoides mccartyi
Tmar_0310, Thermaerobacter marianensis
GBC94369, bacterium HR16
CT0769, Chlorobium tepidum
Pedsa_1350, Pedobacter saltans
Hipma_0235, Hippea maritima
OFV66597, Candidatus Syntrophoarchaeum butanivorans
OFV68847, Candidatus Syntrophoarchaeum caldarius
PTO1403, Picrophilus torridus
WP_071388364, Anaerobacillus alkalidiazotrophicus
WP_098534090, Bacillus cereus
WP_007289038, Thermosinus carboxydivorans
WP_015263024, Desulfitobacterium dichloroeliminans
WP_078664353, Carboxydocella spp.
WP_078809743, Selenihalanaerobacter shriftii
RLG58267, B51_G15
RLG60167, B60_G1
MBGE-E
RLF61483, Thermoplasmata archaeon B12_G9
WP_015591371, Archaeoglobus sulfaticallidus
BMS3Bbin15_00844
IPdc08_00913
MBGE-A
2730022217, JdFR–18
MBGE-C
PKL54702, Candidatus Methanoperedenaceae archaeon
WP_096203609, Candidatus Methanoperedens nitroreducens
RZB29116, Methanosarcinales archaeon Eth-Arch1
HTIA_0615, Halorhabdus tiamatea
Huta_0704, Halorhabdus utahensis
HFX_0966, Haloferax mediterranei
Htur_3181, Haloterrigena turkmenica
Nmag_3253, Natrialba magadii
Caci_8417, Catenulispora acidiphila
Rxyl_1631, Rubrobacter xylanophilus
RradSPS_1572, Rubrobacter radiotolerans
MAB_2137, Mycobacterium abscessus
Strvi_6515, Streptomyces violaceusniger
NQO4_THET8, Thermus thermophilus
NuoD-related
AKB81862, Methanosarcina barkeri
FPOD_METMA, Methanosarcina mazei
WP_015054478, Methanolobus psychrophilus
WP_013193770, Methanohalobium evestigatum
WP_072360385, Methanohalophilus portucalensis
ABK14836, Methanothrix thermoacetophila
WP_014586960, Methanosaeta harundinacea
WP_013720403, Methanothrix soehngenii
PPA79591, ANME–2 cluster archaeon HR1
PXF59645, ANME–2 cluster archaeon E20ANME2
WP_042686462, Methermicoccus shengliensis
BMS3Abin16_00091
MBGE-D
FpoD-related
PAE1582, Pyrobaculum aerophilum
TTX_0215, Thermoproteus tenax
AF_1830, Archaeoglobus fulgidus
Cmaq_0428, Caldivirga maquilingensis
STK_22180, Sulfolobus tokodaii
Msed_1897, Metallosphaera sedula
APE_1422, Aeropyrum pernix
Pyrfu_1964, Pyrolobus fumarii
Kcr_0139, Candidatus Korarchaeum cryptofilum
NTE_02556, Candidatus Nitrososphaera evergladensis
Nmar_0279, Nitrosopumilus maritimus
TGAM_0714, Thermococcus gammatolerans
TK1215, Thermococcus kodakarensis
PF1442, Pyrococcus furiosus
PH1447, Pyrococcus horikoshii
AciM339_0666, Aciduliprofundum sp. strain MAR08-339
Thimo_2412, Thioflavicoccus mobilis
THA_1684, Thermosipho africanus
TCELL_0607, Thermogladius cellulolyticus
Desmu_1004, Desulfurococcus mucosus
Smar_0648, Staphylothermus marinus
MbxL-related
Dester_1185, Desulfurobacterium thermolithotrophum
Igni_0542, Ignicoccus hospitalis
TALC_00212, Thermoplasmatales archaeon BRNA1
MMALV_01980, Candidatus Methanomethylophilus alvus
GSU0743, Geobacter sulfurreducens
Gmet_2597, Geobacter metallireducens
AFE_2153, Acidithiobacillus ferrooxidans
HTH_1154, Hydrogenobacter thermophilus
YE2804, Yersinia enterocolitica
HYFG_ECOLI, Escherichia coli
HYCE_ECOLI, Escherichia coli
CHY_1827, Carboxydothermus hydrogenoformans
Gbem_0069, Geobacter bemidjiensis
Rru_A1425, Rhodospirillum rubrum
MM_2324, Methanosarcina mazei
Mbar_A0148, Methanosarcina barkeri
MCP_0478, Methanocella paludicola
MTH1238, Methanothermobacter thermautotrophicus
Msp_1444, Methanosphaera stadtmanae
MMP1153, Methanococcus maripaludis
MTH398, Methanothermobacter thermautotrophicus
MK0463, Methanopyrus kandleri
MMP1462, Methanococcus maripaludis
Hydrogenases
(EchE, EhaO, HyfG, HycE, CooH)
0.10

Fig. S3

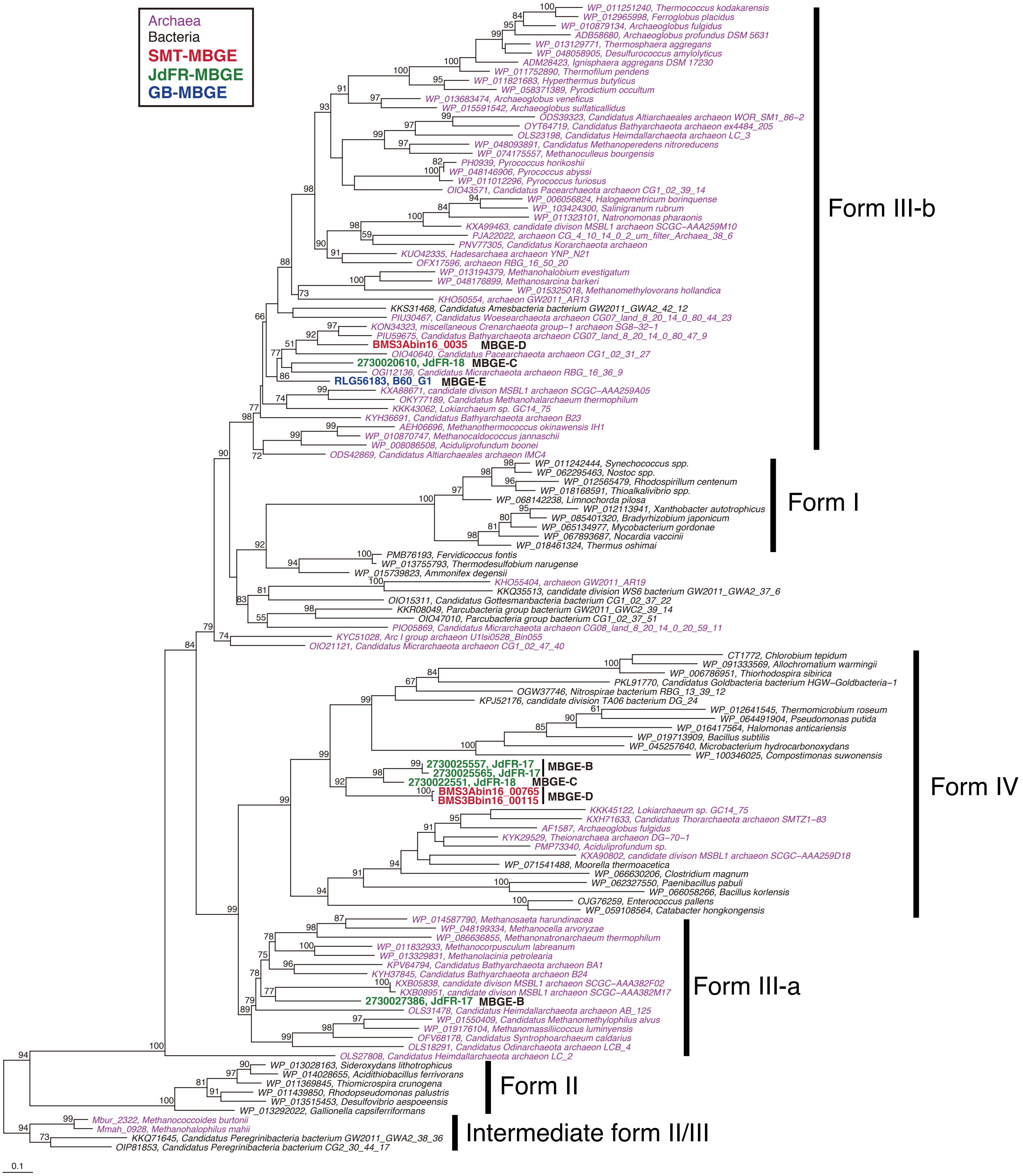

Archaea
Bacteria
SMT-MBGE
JdFR-MBGE
GB-MBGE
WP_011251240, Thermococcus kodakarensis
WP_012965998, Ferroglobus placidus
WP_010879134, Archaeoglobus fulgidus
ADB58680, Archaeoglobus profundus DSM 5631
WP_013129771, Thermosphaera aggregans
WP_048058905, Desulfurococcus amylolyticus
ADM28423, Ignisphaera aggregans DSM 17230
WP_011752890, Thermofilum pendens
WP_011821683, Hyperthermus butylicus
WP_058371389, Pyrodictium occultum
WP_013683474, Archaeoglobus veneficus
WP_015591542, Archaeoglobus sulfaticallidus
ODS39323, Candidatus Altiarchaeales archaeon WOR_SM1_86–2
OYT64719, Candidatus Bathyarchaeota archaeon ex4484_205
OLS23198, Candidatus Heimdallarchaeota archaeon LC_3
WP_048093891, Candidatus Methanoperedens nitroreducens
WP_074175557, Methanoculleus bourgensis
PH0939, Pyrococcus horikoshii
WP_048146906, Pyrococcus abyssi
WP_011012296, Pyrococcus furiosus
OIO43571, Candidatus Pacearchaeota archaeon CG1_02_39_14
WP_006056824, Halogeometricum borinquense
WP_103424300, Salinigranum rubrum
WP_011323101, Natronomonas pharaonis
KXA99463, candidate divison MSBL1 archaeon SCGC–AAA259M10
PJA22022, archaeon CG_4_10_14_0_2_um_filter_Archaea_38_6
PNV77305, Candidatus Korarchaeota archaeon
KUO42335, Hadesarchaea archaeon YNP_N21
OFX17596, archaeon RBG_16_50_20
WP_013194379, Methanohalobium evestigatum
WP_048176899, Methanosarcina barkeri
WP_015325018, Methanomethylovorans hollandica
KHO50554, archaeon GW2011_AR13
KKS31468, Candidatus Amesbacteria bacterium GW2011_GWA2_42_12
PIU30467, Candidatus Woesearchaeota archaeon CG07_land_8_20_14_0_80_44_23
KON34323, miscellaneous Crenarchaeota group–1 archaeon SG8–32–1
PIU59675, Candidatus Bathyarchaeota archaeon CG07_land_8_20_14_0_80_47_9
BMS3Abin16_0035
MBGE-D
OIO40640, Candidatus Pacearchaeota archaeon CG1_02_31_27
2730020610, JdFR-18
MBGE-C
OGI12136, Candidatus Micrarchaeota archaeon RBG_16_36_9
RLG56183, B60_G1
MBGE-E
KXA88671, candidate divison MSBL1 archaeon SCGC–AAA259A05
OKY77189, Candidatus Methanohalarchaeum thermophilum
KKK43062, Lokiarchaeum sp. GC14_75
KYH36691, Candidatus Bathyarchaeota archaeon B23
AEH06696, Methanothermococcus okinawensis IH1
WP_010870747, Methanocaldococcus jannaschii
WP_008086508, Aciduliprofundum boonei
ODS42869, Candidatus Altiarchaeales archaeon IMC4
Form III-b
WP_011242444, Synechococcus spp.
WP_062295463, Nostoc spp.
WP_012565479, Rhodospirillum centenum
WP_018168591, Thioalkalivibrio spp.
WP_068142238, Limnochorda pilosa
WP_012113941, Xanthobacter autotrophicus
WP_085401320, Bradyrhizobium japonicum
WP_065134977, Mycobacterium gordonae
WP_067893687, Nocardia vaccinii
WP_018461324, Thermus oshimai
Form I
PMB76193, Fervidicoccus fontis
WP_013755793, Thermodesulfobium narugense
WP_015739823, Ammonifex degensii
KHO55404, archaeon GW2011_AR19
KKQ35513, candidate division WS6 bacterium GW2011_GWA2_37_6
OIO15311, Candidatus Gottesmanbacteria bacterium CG1_02_37_22
KKR08049, Parcubacteria group bacterium GW2011_GWC2_39_14
OIO47010, Parcubacteria group bacterium CG1_02_37_51
PIO05869, Candidatus Micrarchaeota archaeon CG08_land_8_20_14_0_20_59_11
KYC51028, Arc I group archaeon U1lsi0528_Bin055
OIO21121, Candidatus Micrarchaeota archaeon CG1_02_47_40
CT1772, Chlorobium tepidum
WP_091333569, Allochromatium warmingii
WP_006786791, Thiorhodospira sibirica
PKL91770, Candidatus Goldbacteria bacterium HGW–Goldbacteria–1
OGW37746, Nitrospirae bacterium RBG_13_39_12
KPJ52176, candidate division TA06 bacterium DG_24
WP_012641545, Thermomicrobium roseum
WP_064491904, Pseudomonas putida
WP_016417564, Halomonas anticariensis
WP_019713909, Bacillus subtilis
WP_045257640, Microbacterium hydrocarbonoxydans
WP_100346025, Compostimonas suwonensis
2730025557, JdFR-17
2730025565, JdFR-17
MBGE-B
2730022551, JdFR-18
MBGE-C
BMS3Abin16_00765
BMS3Bbin16_00115
MBGE-D
KKK45122, Lokiarchaeum sp. GC14_75
KXH71633, Candidatus Thorarchaeota archaeon SMTZ1–83
AF1587, Archaeoglobus fulgidus
KYK29529, Theionarchaea archaeon DG–70–1
PMP73340, Aciduliprofundum sp.
KXA90802, candidate divison MSBL1 archaeon SCGC–AAA259D18
WP_071541488, Moorella thermoacetica
WP_066630206, Clostridium magnum
WP_062327550, Paenibacillus pabuli
WP_066058266, Bacillus korlensis
OJG76259, Enterococcus pallens
WP_059108564, Catabacter hongkongensis
Form IV
WP_014587790, Methanosaeta harundinacea
WP_048199334, Methanocella arvoryzae
WP_086636855, Methanonatronarchaeum thermophilum
WP_011832933, Methanocorpusculum labreanum
WP_013329831, Methanolacinia petrolearia
KPV64794, Candidatus Bathyarchaeota archaeon BA1
KYH37845, Candidatus Bathyarchaeota archaeon B24
KXB05838, candidate divison MSBL1 archaeon SCGC–AAA382F02
KXB08951, candidate divison MSBL1 archaeon SCGC–AAA382M17
2730027386, JdFR-17
MBGE-B
OLS31478, Candidatus Heimdallarchaeota archaeon AB_125
WP_01550409, Candidatus Methanomethylophilus alvus
WP_019176104, Methanomassiliicoccus luminyensis
OFV68178, Candidatus Syntrophoarchaeum caldarius
OLS18291, Candidatus Odinarchaeota archaeon LCB_4
Form III-a
OLS27808, Candidatus Heimdallarchaeota archaeon LC_2
WP_013028163, Sideroxydans lithotrophicus
WP_014028655, Acidithiobacillus ferrivorans
WP_011369845, Thiomicrospira crunogena
WP_011439850, Rhodopseudomonas palustris
WP_013515453, Desulfovibrio aespoeensis
WP_013292022, Gallionella capsiferriformans
Form II
Mbur_2322, Methanococcoides burtonii
Mmah_0928, Methanohalophilus mahii
KKQ71645, Candidatus Peregrinibacteria bacterium GW2011_GWA2_38_36
OIP81853, Candidatus Peregrinibacteria bacterium CG2_30_44_17
Intermediate form II/III
0.1

Fig. S4

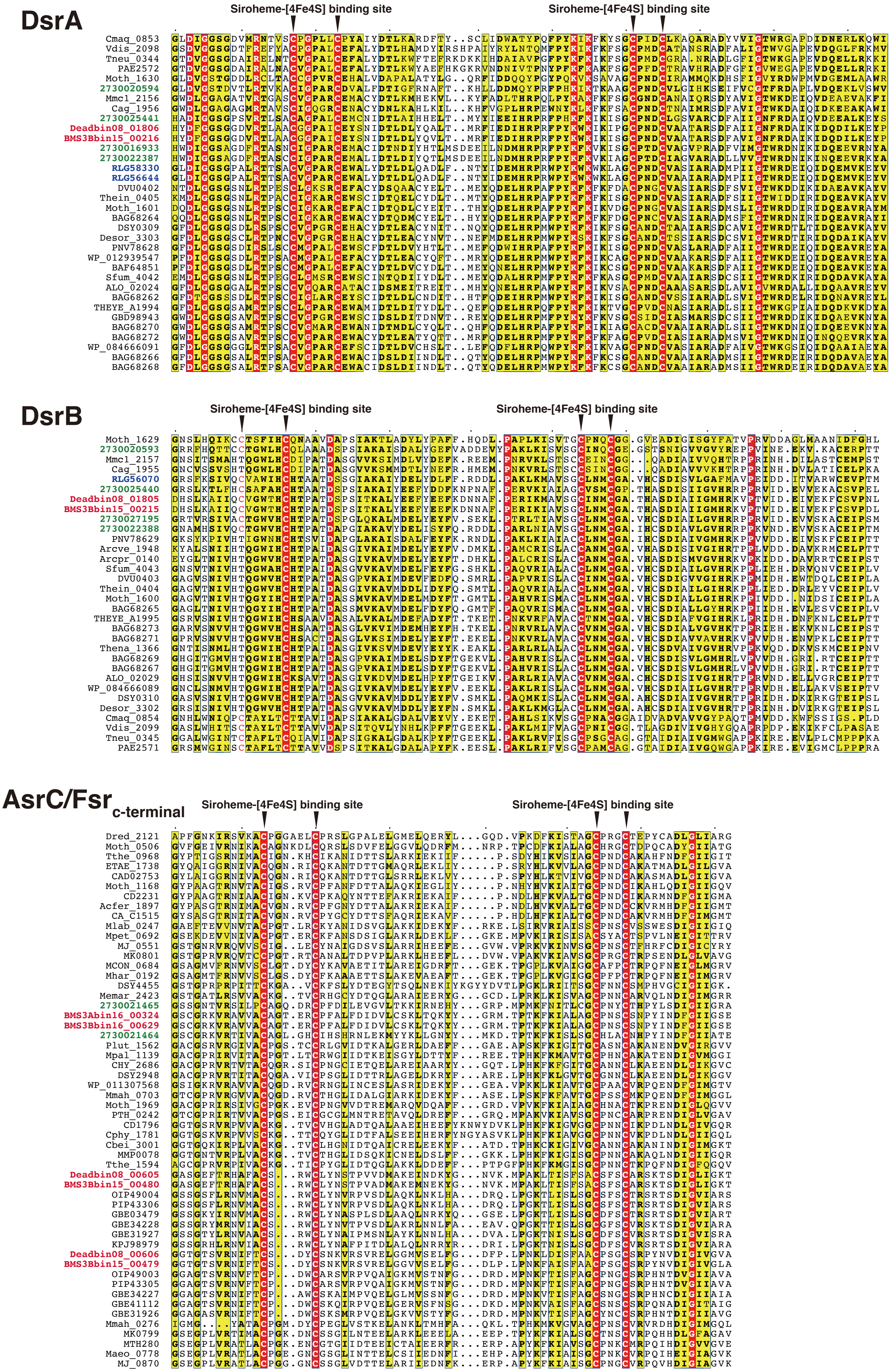

DsrA
Siroheme-[4Fe4S] binding site
Siroheme-[4Fe4S] binding site
DsrB
Siroheme-[4Fe4S] binding site
Siroheme-[4Fe4S] binding site
AsrC/Fsr c-terminal
Siroheme-[4Fe4S] binding site
Siroheme-[4Fe4S] binding site

Fig. S5

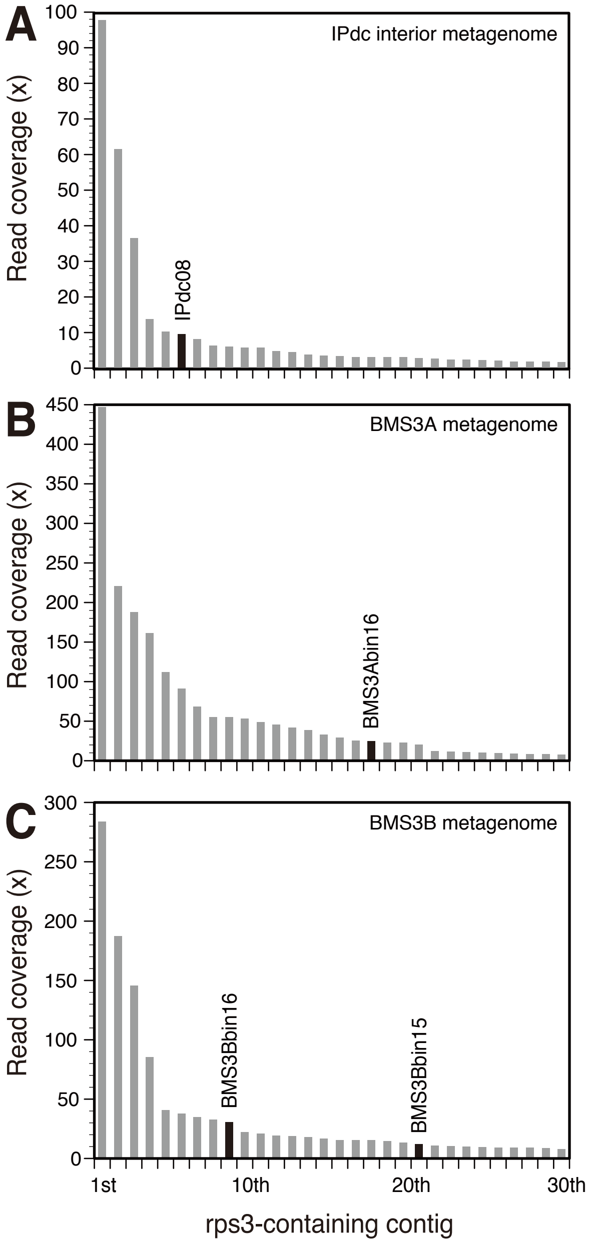
A
IPdc interior metagenome
Read coverage (x)
IPdc08
B
BMS3A metagenome
BMS3Abin16
C
BMS3B metagenome
BMS3Bbin16
BMS3Bbin15
1st
10th
20th
30th
rps3-containing contig

Fig. S6

Table S1. List of CDSs used in Fig. 2

KEGG or COG	Gene/sub unit name	Inactive chimney	Subseafloor massive sulfide	Subseafloor hydrothermal fluid			Subseafloor massive sulfide		Hydrothermal sediment	
		MBGE-A		MBGE-B		MBGE-C	MBGE-D		MBGE-E	
		IPdc08	BMS3Bbin15	JdFR-16	JdFR-17	JdFR-18	BMS3Abin16	BMS3Bbin16	B51_G15	B60_G1
**Metabolism**										
**Sulfur metabolism**										
**Sulfate reduction, sulfate => sulfide**										
sulfate adenylyltransferase [EC:2.7.7.4]										
K00958	*sat*					2730021466	BMS3Abin16_00323	BMS3Bbin16_00630		
adenylylsulfate reductase [EC:1.8.99.2]										
K00394	*aprA*					2730021468	BMS3Abin16_00321	BMS3Bbin16_00632		
K00395	*aprB*					2730021467	BMS3Abin16_00322	BMS3Bbin16_00631		
anaerobic sulfite reductase										
COG2221	*asrC*	IPdc08_00605, IPdc08_00606	BMS3Bbin15_00479, BMS3Bbin15_00480	2730016886		2730021465, 2730021464	BMS3Abin16_00324	BMS3Bbin16_00629		
dissimilatory sulfite reductase [EC:1.8.99.5]										
K11180	*dsrA*	IPdc08_01806	BMS3Bbin15_00216	2730016933	2730025441	2730022387, 2730020594			RLG56644, RLG56066	RLG58330
K11181	*dsrB*	IPdc08_01805	BMS3Bbin15_00215		2730025440, 2730027195	2730022388, 2730020593			RLG56070	RLG58602
K11179	*dsrC*	IPdc08_00964	BMS3Bbin15_00093	2730017136, 2730015767	2730027622, 2730025717	2730021846				RLG59165
COG0247	*dsrK*		BMS3Bbin15_01898	2730016247	2730027829, 2730026763				RLG55608	RLG59168
COG2181	*dsrM*		BMS3Bbin15_01899	2730016248	2730027830, 2730026764	2730021851				RLG58994
adenylylsulfate kinase [EC:2.7.1.25]										
K00860	*cysC*						BMS3Abin16_00597			
phosphoadenosine phosphosulfate reductase [EC:1.8.4.8 1.8.4.10]										
K00390	*cysH*		BMS3Bbin15_00831, BMS3Bbin15_01080	2730016603	2730027551	2730021875, 2730021704, 2730020541	BMS3Abin16_01077			RLG56768
**Thiosulfate reduction, thiosulfate => sulfide**										
thiosulfate sulfurtransferase [EC:2.8.1.1 2.8.1.2]										
K01011	*tst*	IPdc08_01662	BMS3Bbin15_01981, BMS3Bbin15_01982	2730017019	2730026871, 2730026109			BMS3Bbin16_00574		RLG58985
**Tetrathionate reduction, tetrathionate => thiosulfate**										
K08357	*ttrA*		BMS3Bbin15_01571				BMS3Abin16_00405			
K08358	*ttrB*		BMS3Bbin15_01570				BMS3Abin16_00406			
**Sulfide oxidation, sulfide => polysulfide**										
sulfide:quinone oxidoreductase [EC:1.8.5.4]										
K17218	*sqr*			2730016033	2730025858					
**Hydrogen metabolism**										
**Hydrogen oxidation/production**										
Group 1 [NiFe] Hydrogenase										
K06281	*Large*					2730020743	BMS3Abin16_01391	BMS3Bbin16_01271	RLG56988, RLG55404	
K06282	*Small*					2730020741	BMS3Abin16_01390	BMS3Bbin16_01270	RLG56989, RLG55405	
-	*Cyt b*					2730020742	BMS3Abin16_01392	BMS3Bbin16_01272	RLG56987	
Group 1/2 [NiFe] Hydrogenase										
-	*Large*		BMS3Bbin15_00500	2730016502, 2730016450	2730025648, 2730027434					
-	*Small*		BMS3Bbin15_00501	2730016451	2730025649, 2730027435					
Group 3 [NiFe] Hydrogenase										
K14126	*Large*							BMS3Bbin16_00402		
K14128	*Small*							BMS3Bbin16_00401		
Group 4 [NiFe] Hydrogenase										
COG3261	*Large*	IPdc08_01109	BMS3Bbin15_00881							
COG3260	*Small*	IPdc08_01108	BMS3Bbin15_00880							
**Nitrogen metabolism**										
**Nitrogen fixation, N2 => ammonia**										
nitrogenase [EC:1.18.6.1]										
K02588	*nifH*	IPdc08_00497	BMS3Bbin15_01846				BMS3Abin16_01217	BMS3Bbin16_01169		
K02586	*nifD*	IPdc08_00493	BMS3Bbin15_01850				BMS3Abin16_01221	BMS3Bbin16_01277		
K02591	*nifK*	IPdc08_00492	BMS3Bbin15_01851				BMS3Abin16_01222	BMS3Bbin16_01278		
K02585	*nifB*	IPdc08_00494	BMS3Bbin15_01849				BMS3Abin16_01220	BMS3Bbin16_01166		
K02589	*nifI1*	IPdc08_00496	BMS3Bbin15_01847				BMS3Abin16_01218	BMS3Bbin16_01168		
K02590	*nifI2*	IPdc08_00495	BMS3Bbin15_01848				BMS3Abin16_01219	BMS3Bbin16_01167		
**Denitrification, nitrate => N2**										
nitric oxide reductase [EC:1.7.2.5]										
K04561	*norB*					2730020591				
K02305	*norC*					2730020592				
nitrous-oxide reductase [EC:1.7.2.4]										
K00376	*nosZ*				2730026177					
**Dissimilatory nitrate reduction, nitrate => ammonia**										
nitrate reductase / nitrite oxidoreductase [EC:1.7.5.1 1.7.99.4]										
K00370	*narG*				2730025434	2730021156				
K00371	*narH*				2730025435	2730021153				
K00373	*narJ*				2730025436					
K00374	*narI*				2730025437					
periplasmic nitrate reductase NapA [EC:1.7.99.4]										
K02567	*napA*			2730016772	2730025251	2730021142				
K02570	*napD*				2730027786, 2730026600	2730021141				
K02573	*napG*				2730025249					
K02574	*napH*				2730026599, 2730027787					
**cyanate lysis, cyanate => ammonia**										
cyanate lyase [EC:4.2.1.104]										
K01725	*cynS*		BMS3Bbin15_01379					Bin08_01786		
**Carbon metabolism**										
**Archaeal Wood-Ljungdahl pathway**										
formate dehydrogenase [EC:1.2.1.43]										
K00123	*fdoG*			2730017096	2730025969	2730020745			RLG57524	RLG58050
K00124	*fdoH*			2730017097	2730025968	2730020746			RLG57523, RLG57259	RLG58656

formylmethanofuran--tetrahydromethanopterin N-formyltransferase [EC:2.3.1.101]										
K00672	*ftr*	IPdc08_00212, IPdc08_01114	BMS3Bbin15_00984, BMS3Bbin15_01661	2730015980	2730027711, 2730026057	2730022359, 2730020652	BMS3Abin16_00005, BMS3Abin16_00725	BMS3Bbin16_00049, BMS3Bbin16_00470	RLG55426	RLG59664
methenyltetrahydromethanopterin cyclohydrolase [EC:3.5.4.27]										
K01499	*mch*	IPdc08_01668	BMS3Bbin15_01803			2730022046	BMS3Abin16_00425		RLG54341	
methylenetetrahydromethanopterin dehydrogenase [EC:1.5.98.1]										
K00319	*mtd*	IPdc08_01513	BMS3Bbin15_00468		2730025598	2730021346			RLG57835	RLG60269
5,10-methylenetetrahydromethanopterin reductase [EC:1.5.98.2]										
K00320	*mer*	IPdc08_00314, IPdc08_00315	BMS3Bbin15_01814, BMS3Bbin15_01815			2730021183, 2730020646, 2730022504, 2730022503		BMS3Bbin16_01245, BMS3Bbin16_01246		
formylmethanofuran dehydrogenase [EC:1.2.7.12]										
K00200	*fwdA*	IPdc08_01603	BMS3Bbin15_01589	2730016924	2730026415	2730021643	BMS3Abin16_00971	BMS3Bbin16_00465	RLG56330	RLG59547
K00201	*fwdB*	IPdc08_01604	BMS3Bbin15_01588		2730026416	2730021642	BMS3Abin16_00970	BMS3Bbin16_00466	RLG58283	RLG58509
K00202	*fwdC*	IPdc08_01602	BMS3Bbin15_01590	2730016923	2730026414	2730021644	BMS3Abin16_00972		RLG56331	RLG59548
K00203	*fwdD*	IPdc08_01605	BMS3Bbin15_01587		2730026417	2730021641	BMS3Abin16_00969	BMS3Bbin16_01131	RLG58284	RLG58508
K00205	*fwdF*	IPdc08_01607	BMS3Bbin15_01585		2730026419	2730021639	BMS3Abin16_00581	BMS3Bbin16_01129	RLG57836	RLG60256
K11260	*fwdG*		BMS3Bbin15_01534	2730016427	2730027571, 2730026958	2730021907	BMS3Abin16_01032		RLG58285	
K11261	*fwdE*			2730016882, 2730016780	2730026721, 2730025501, 2730025259				RLG56747	
formylmethanofuran dehydrogenase [EC:1.2.7.12]										
K13812	*fae-hps*		BMS3Bbin15_01576		2730026756	2730021516		BMS3Bbin16_00463, BMS3Bbin16_00464		
tetrahydromethanopterin S-methyltransferase subunit A [EC:2.1.1.86]										
K00577	*mtrA*	IPdc08_00727	BMS3Bbin15_01277				BMS3Abin16_01813			
K00584	*mtrH*			2730016920		2730020980, 2730020978				
heterodisulfide reductase [EC:1.8.98.1]										
K03388	*hdrA*	IPdc08_00747		2730017380, 2730016799	2730025372, 2730027164	2730021679, 2730020790, 2730020788	BMS3Abin16_00428, BMS3Abin16_00849, BMS3Abin16_00851, BMS3Abin16_01381, BMS3Abin16_01382	BMS3Bbin16_00109, BMS3Bbin16_00399, BMS3Bbin16_00806, BMS3Bbin16_01223	RLG57838, RLG57113, RLG57115, RLG56619, RLG56329, RLG55337, RLG57059, RLG55725	RLG60262, RLG59546, RLG59340
K03389	*hdrB*	IPdc08_00599	BMS3Bbin15_00265, BMS3Bbin15_00340, BMS3Bbin15_01422	2730017249, 2730017156, 2730016549, 2730015752	2730026941, 2730026878, 2730026794, 2730026506, 2730026470	2730022197, 2730021085	BMS3Abin16_00749, BMS3Abin16_00750, BMS3Abin16_01600		RLG55339, RLG56013, RLG55727	
K03390	*hdrC*	IPdc08_00172, IPdc08_00600	BMS3Bbin15_01423	2730017157, 2730016548	2730026940, 2730026877, 2730026795	2730022198, 2730021086	BMS3Abin16_00751, BMS3Abin16_01601		RLG57968, RLG56012	RLG59108
K08264	*hdrD*				2730027667	2730022093, 2730020998				
[methyl-Co(III) methanol-specific corrinoid protein]:coenzyme M methyltransferase [EC:2.1.1.246]										
K14080	*mtaA*				2730027678, 2730027674	2730022238, 2730020975, 2730020973				
trimethylamine---corrinoid protein Co-methyltransferase [EC:2.1.1.250]										
K14083	*mttB*					2730021299, 2730021298				
K14084	*mttC*					2730021300				
dimethylamine---corrinoid protein Co-methyltransferase [EC:2.1.1.249]										
K16178	*mtbB*					2730021320, 2730021319, 2730020721, 2730020720				
K16179	*mtbC*					2730021321				
methylamine---corrinoid protein Co-methyltransferase [EC:2.1.1.248]										
K16176	*mtmB*					2730021313, 2730021312, 2730020894				
acetyl-CoA decarbonylase/synthase complex subunit alpha [EC:1.2.7.4, EC:2.1.1.245]										
K00192	*cdhA*	IPdc08_01043	BMS3Bbin15_00135		2730027556, 2730025989	2730020794	BMS3Abin16_00059		RLG55634	
K00193	*cdhC*	IPdc08_00130	BMS3Bbin15_01494, BMS3Bbin15_01531	2730017220, 2730016795	2730027539, 2730027501, 2730026574, 2730025724	2730021910	BMS3Abin16_00061		RLG58288	
K00194	*cdhD*	IPdc08_01196	BMS3Bbin15_01533	2730016428	2730027570, 2730026959	2730021908	BMS3Abin16_01878	BMS3Bbin16_00981	RLG58286	
K00195	*cdhB*	IPdc08_01044	BMS3Bbin15_00134			2730020795	BMS3Abin16_00060		RLG58289	
K00197	*cdhE*	IPdc08_01198	BMS3Bbin15_01535	2730016426	2730027572, 2730026957	2730021906, 2730022548	BMS3Abin16_01407		RLG58291	RLG58507
K00198	*cooS*		BMS3Bbin15_00707, BMS3Bbin15_01658	2730016113, 2730015846	2730027368, 2730025767		BMS3Abin16_00069			
**Calvin–Benson–Bassham cycle**										
ribulose-bisphosphate carboxylase [EC:4.1.1.39]										
K01601	*rbcL*				2730027386, 2730025565, 2730025557	2730022551, 2730020610	BMS3Abin16_00352, BMS3Abin16_00765	BMS3Bbin16_00115	RLG56183	
**Glycolysis / Gluconeogenesis**										
phosphomannomutase / phosphoglucomutase [EC:5.4.2.8 5.4.2.2]										
K15778	*pgm*	IPdc08_01274	BMS3Bbin15_00825	2730015762	2730026496, 2730026459	2730022670		BMS3Bbin16_00994	RLG55663	
glucokinase [EC:2.7.1.2; EC:2.7.1.146; EC:2.7.1.147]										
K00918	*pfkC*	IPdc08_01320	BMS3Bbin15_00298	2730016096, 2730015965						
K00845	*glk*						BMS3Abin16_00803		RLG56185	
glucose-6-phosphate isomerase, archaeal [EC:5.3.1.9]										
K06859	*pgiI*					2730022537				
fructose 1,6-bisphosphate aldolase/phosphatase [EC:4.1.2.13 3.1.3.11]										
K03841	*fbp*						BMS3Abin16_00867	BMS3Bbin16_00482		
K01622	*fbap*	IPdc08_01490	BMS3Bbin15_01233		2730026903, 2730025397	2730022730			RLG56189	
phosphofructokinase [EC:2.7.1.11; EC:2.7.1.90]										
K21071	*pfk*						BMS3Abin16_00127	BMS3Bbin16_00996		

K00918	*pfkC*	IPdc08_01320	BMS3Bbin15_00298	2730016096, 2730015965						
triosephosphate isomerase (TIM) [EC:5.3.1.1]										
K01803	*tpi*	IPdc08_01237	BMS3Bbin15_00310		2730026904, 2730025396	2730022731	BMS3Abin16_01669		RLG56187, RLG55770	
glyceraldehyde 3-phosphate dehydrogenase [EC:1.2.1.12]										
K00134	*gapA*	IPdc08_01487	BMS3Bbin15_01236			2730022728	BMS3Abin16_01167	BMS3Bbin16_00270	RLG53523	RLG58494
phosphoglycerate kinase [EC:2.7.2.3]										
K00927	*pgk*	IPdc08_00796	BMS3Bbin15_00870	2730017359	2730027652, 2730026977	2730021965	BMS3Abin16_01513	BMS3Bbin16_00722	RLG56020	
2,3-bisphosphoglycerate-dependent phosphoglycerate mutase [EC:5.4.2.11; EC:5.4.2.12]										
K01834	*gpmA*						BMS3Abin16_00126			
K15635	*apgM*	IPdc08_00318	BMS3Bbin15_00234, BMS3Bbin15_01811		2730027644, 2730026953, 2730025974	2730022669, 2730021990	BMS3Abin16_00130, BMS3Abin16_01801	BMS3Bbin16_00707, BMS3Bbin16_00993	RLG57916, RLG55662	RLG60017
enolase [EC:4.2.1.11]										
K01689	*eno*	IPdc08_01142	BMS3Bbin15_01941	2730016211, 2730015789	2730026165, 2730025213	2730022720	BMS3Abin16_00952	BMS3Bbin16_00157		RLG58828
pyruvate, water dikinase [EC:2.7.9.2]										
K01007	*pps*	IPdc08_01275	BMS3Bbin15_00826			2730021384	BMS3Abin16_00220	BMS3Bbin16_00393	RLG57978	RLG59822
phosphoenolpyruvate carboxylase [EC:4.1.1.31]										
K01595	*ppc*	IPdc08_00733	BMS3Bbin15_00956	2730017216, 2730016791	2730027544, 2730026578	2730021377				
malate dehydrogenase [EC:1.1.1.38]										
K00027	*mae*	IPdc08_00457	BMS3Bbin15_00521							
pyruvate dehydrogenase [EC:1.2.4.1; EC:2.3.1.12; EC:1.8.1.4]										
K00161	*pdhA*					2730020850				
K00162	*pdhB*					2730020849				
K00627	*pdhC*					2730020848				
K00382	*pdhD*		BMS3Bbin15_00594				BMS3Abin16_01385			
pyruvate ferredoxin oxidoreductase [EC:1.2.7.1]										
K00169	*porA*	IPdc08_00210	BMS3Bbin15_01780		2730025820	2730020654	BMS3Abin16_01656	BMS3Bbin16_01262	RLG57611	RLG59147
K00170	*porB*	IPdc08_00211							RLG57610	RLG59148
K00171	*porD*	IPdc08_00209							RLG57612	RLG59146
K00172	*porG*	IPdc08_00208							RLG57613	RLG59145
2-oxoglutarate/2-oxoacid ferredoxin oxidoreductase subunit alpha [EC:1.2.7.3 1.2.7.11]										
K00174	*oforA*	IPdc08_01564, IPdc08_01902	BMS3Bbin15_00273, BMS3Bbin15_01831	2730016282, 2730016258	2730026732, 2730026717					
K00175	*oforB*	IPdc08_01565, IPdc08_01903	BMS3Bbin15_00272, BMS3Bbin15_01832	2730016281, 2730016257	2730026731, 2730026716					
**Acetate, lactate, pyruvate synthetase/licase**										
acetate---CoA ligase (ADP-forming) subunit beta [EC:6.2.1.13]										
K01905	*acdA*	IPdc08_00372	BMS3Bbin15_00122			2730022123				
K22224	*acdB*					2730022124			RLG57410, RLG53602	
acetyl-CoA synthetase [EC:6.2.1.1]										
K01895	*acs*		BMS3Bbin15_00240		2730027901	2730022520	BMS3Abin16_00151, BMS3Abin16_01640	BMS3Bbin16_00235	RLG58443	RLG58843, RLG56150
L-lactate dehydrogenase [EC:1.1.1.27]										
K00016	*ldh*					2730021554	BMS3Abin16_00364			
pyruvate carboxylase [EC:6.4.1.1]										
K01959	*pycA*	IPdc08_01815	BMS3Bbin15_01712	2730016699	2730027411, 2730026154	2730020970	BMS3Abin16_01408		RLG55464	RLG58884
K01960	*pycB*	IPdc08_00818	BMS3Bbin15_00497	2730015930	2730027913, 2730027723	2730020972	BMS3Abin16_00667	BMS3Bbin16_00079	RLG55465, RLG56334.1	RLG58885
**TCA cycle**										
aconitate hydratase [EC:4.2.1.3]										
K01681	*aco*						BMS3Abin16_00693	BMS3Bbin16_00257		
isocitrate dehydrogenase [EC:1.1.1.42]										
K00031	*icd*					2730021559			RLG57798, RLG56648	
2-oxoglutarate/2-oxoacid ferredoxin oxidoreductase subunit alpha [EC:1.2.7.3 1.2.7.11]										
K00174	*korA*	IPdc08_01564, IPdc08_01902	BMS3Bbin15_00273, BMS3Bbin15_01831	2730016282, 2730016258	2730026732, 2730026717					
K00175	*korB*	IPdc08_01565, IPdc08_01903	BMS3Bbin15_00272, BMS3Bbin15_01832	2730016281, 2730016257	2730026731, 2730026716					
K00177	*korC*	IPdc08_01904	BMS3Bbin15_00271		2730026407					
K00176	*korD*	IPdc08_01900	BMS3Bbin15_00274	2730017181, 2730016758	2730025635					
succinyl-CoA synthetase [EC:6.2.1.5]										
K01902	*sucD*	IPdc08_00216	BMS3Bbin15_00976	2730016003	2730027648, 2730026973	2730021388				
K01903	*sucC*	IPdc08_00215	BMS3Bbin15_00975		2730027647, 2730026972	2730021389				
succinate dehydrogenase [EC:1.3.5.1 1.3.5.4]										
K00239	*sdhA*					2730020609				
K00240	*sdhB*					2730020607				
K00241	*sdhC*					2730020608			RLG57967	RLG59107
fumarate hydratase [EC:4.2.1.2]										
K01677	*fumA*	IPdc08_01609	BMS3Bbin15_01583		2730027183, 2730025274				RLG54272	
K01678	*fumB*	IPdc08_01608	BMS3Bbin15_01584	2730015882	2730027184, 2730025273				RLG55300, RLG56405	
K01679	*fumC*						BMS3Abin16_01327			
**Pentose phosphate pathway (Pentose phosphate cycle)**										
3-hexulose-6-phosphate synthase / 6-phospho-3-hexuloisomerase [EC:4.1.2.43 5.3.1.27]/bifunctional enzyme Fae/Hps [EC:4.2.1.147 4.1.2.43]										
K08094	*hxlB*	IPdc08_01518	BMS3Bbin15_01406			2730021481	BMS3Abin16_00709		RLG56732	
K13831	*hps-phi*	IPdc08_01040	BMS3Bbin15_01309	2730016049	2730026555, 2730025878	2730021515	BMS3Abin16_00580	BMS3Bbin16_00656, BMS3Bbin16_01126	RLG56149	
K13812	*fae-hps*	IPdc08_01228	BMS3Bbin15_01576		2730026756	2730021516		BMS3Bbin16_00463, BMS3Bbin16_00464		
ribulose-phosphate 3-epimerase [EC:5.1.3.1]										
K01783	*rpe*						BMS3Abin16_01036	BMS3Bbin16_00625		
ribose 5-phosphate isomerase A [EC:5.3.1.6]										
K01807	*rpiA*	IPdc08_01653	BMS3Bbin15_01619	2730015938		2730021177	BMS3Abin16_01180	BMS3Bbin16_00685	RLG57552	
transketolase [EC:2.2.1.1]										
K00615	*tkt*						BMS3Abin16_00801			
ribokinase [EC:2.7.1.15]										
K00852	*rbsK*	IPdc08_00135	BMS3Bbin15_01972	2730015884	2730027189, 2730025272	2730022492, 2730021636	BMS3Abin16_01479		RLG56565	RLG59408

ribose-phosphate pyrophosphokinase [EC:2.7.6.1]										
K00948	*prps*	IPdc08_00256	BMS3Bbin15_01606			2730022192	BMS3Abin16_01530	BMS3Bbin16_00550	RLG58250	RLG59244
**Peptide degradation**										
Peptidase family C56										
K05520	*pfpI*				2730025615				RLG56337, RLG57354	
Peptidase family M24										
K01265	*map*	IPdc08_00332	BMS3Bbin15_00597	2730016732	2730026892, 2730025823	2730021067	BMS3Abin16_01556	BMS3Bbin16_00313	RLG57905, RLG57146, RLG56859, RLG56769.1	RLG59260,
Peptidase family M48										
K03799	*htpX*	IPdc08_00900		2730017418, 2730015811	2730027449, 2730026397	2730021030			RLG58105	
Peptidase family M54										
K06974	*amzA*	IPdc08_01634	BMS3Bbin15_00269, BMS3Bbin15_01959	2730016959, 2730016230, 2730015812, 2730015744	2730026514, 2730026398, 2730025448	2730022735			RLG57721, RLG57803	RLG58860
Peptidase family S8 (extracellular)										
K17734	*aprX*				**2730026073***	**2730021371***				
Peptidase family S16										
K01338	*lon*	IPdc08_00031					BMS3Abin16_00256	BMS3Bbin16_01203		
K04076	*lonB*	IPdc08_00174, IPdc08_00569	BMS3Bbin15_01757			2730022193	BMS3Abin16_01529	BMS3Bbin16_00551	RLG58249	RLG59245
Peptidase family S49										
K04773	*sppA*	IPdc08_00959	BMS3Bbin15_00088		2730025617	2730022409, 2730020684	BMS3Abin16_00336	BMS3Bbin16_00290	RLG58115	RLG58627
Peptidase family T1										
K03432	*psmA*	IPdc08_00985	BMS3Bbin15_01684	2730016541	2730025834, 2730025418	2730021804		BMS3Bbin16_00855	RLG58042	RLG59304
K03433	*psmB*	IPdc08_00739	BMS3Bbin15_00958	2730016580	2730027852, 2730026428	2730020952	BMS3Abin16_00643	BMS3Bbin16_00296		RLG58562
Peptidase family U32										
K08303	-	IPdc08_00888	BMS3Bbin15_00718				BMS3Abin16_00422, BMS3Abin16_00423	BMS3Bbin16_00441, BMS3Bbin16_00442		
Peptidase family U62										
K03592	*pmbA*	IPdc08_00176				2730020923	BMS3Abin16_01527		RLG58247	RLG59514
K03568	*tldD*	IPdc08_00175				2730020924	BMS3Abin16_01528		RLG58248	RLG59513
glutamate dehydrogenase (NAD(P)+) [EC:1.4.1.3]										
K00261	*gdhA*					2730021713		BMS3Bbin16_01196		
aspartate ammonia-lyase [EC:4.3.1.1]										
K01744	*aspA*					2730021226				
aspartate aminotransferase [EC:2.6.1.1]										
K00812	*aspB*	IPdc08_00812, IPdc08_01269	BMS3Bbin15_00612, BMS3Bbin15_01479	2730017145, 2730015776	2730027868, 2730026697	2730022316, 2730020901	BMS3Abin16_00846, BMS3Abin16_00888	BMS3Bbin16_00041, BMS3Bbin16_00264, BMS3Bbin16_01209	RLG57014	RLG58903
branched-chain amino acid aminotransferase [EC:2.6.1.42]										
K00826	*ilvE*	IPdc08_01724	BMS3Bbin15_01403	2730016711	2730025803	2730021451	BMS3Abin16_00989		RLG58434	
aminotransferase [EC:2.6.1.-]										
K10907	-					2730021087	BMS3Abin16_00659		RLG58474	RLG58735
alcohol dehydrogenase, propanol-preferring [EC:1.1.1.1]										
K13953	*adhP*					2730021303				
aldehyde:ferredoxin oxidoreductase [EC:1.2.7.5]										
K03738	*aor*	IPdc08_00366, IPdc08_00837	BMS3Bbin15_00116, BMS3Bbin15_00202		2730027693, 2730026133	2730022442, 2730020898, 2730020718			RLG58320, RLG58263, RLG53667	RLG60163, RLG59352
**Energy metabolism**										
**Archaeal-type NADH:quinone oxidoreductase**										
NADH-quinone oxidoreductase [EC:1.6.5.3] / F420H2 dehydrogenase [EC:1.5.98.3]										
K00330	*nuoA*		BMS3Bbin15_00841			2730022221				
K00331	*nuoB*	IPdc08_00915	BMS3Bbin15_00842		2730025924	2730022220	BMS3Abin16_00825	BMS3Bbin16_00351	RLG58265	RLG60165
K00332	*nuoC*	IPdc08_00914	BMS3Bbin15_00843			2730022219			RLG58266	RLG60166
K00333	*nuoD*	IPdc08_00913	BMS3Bbin15_00844			2730022217	BMS3Abin16_00091		RLG58267	RLG60167
K00337	*nuoH*	IPdc08_00912	BMS3Bbin15_00845			2730022216	BMS3Abin16_00092	BMS3Bbin16_00353	RLG58268	RLG60168
K00338	*nuoI*	IPdc08_00911	BMS3Bbin15_00846			2730022215			RLG58274	RLG60174
K00339	*nuoJ*	IPdc08_00910	BMS3Bbin15_00847							
K00340	*nuoK*	IPdc08_00909	BMS3Bbin15_00848				BMS3Abin16_00095	BMS3Bbin16_00356		
K00341	*nuoL*	IPdc08_00908	BMS3Bbin15_00849							
K00342	*nuoM*	IPdc08_00907	BMS3Bbin15_00850				BMS3Abin16_00723			
K00343	*nuoN*	IPdc08_00906	BMS3Bbin15_00851				BMS3Abin16_00722			
**Ferredoxin:NAD+ oxidoreductase**										
electron transport complex protein										
K03615	*rnfC*						BMS3Abin16_01608			
K03614	*rnfD*						BMS3Abin16_01609	BMS3Bbin16_00241		
K03612	*rnfG*						BMS3Abin16_01610	BMS3Bbin16_00242		
K03613	*rnfE*						BMS3Abin16_01611	BMS3Bbin16_00243		
K03617	*rnfA*						BMS3Abin16_01612	BMS3Bbin16_01094		
K03616	*rnfB*						BMS3Abin16_00023	BMS3Bbin16_01093		
**Coenzyme B:coenzyme M:methanophenazine oxidoreductase**										
heterodisulfide reductase [EC:1.8.98.1]										
K03388	*hdrA*	IPdc08_00747		2730017380, 2730016799	2730025372, 2730027164	2730021679, 2730020790, 2730020788	BMS3Abin16_00428, BMS3Abin16_00849, BMS3Abin16_00851, BMS3Abin16_01381, BMS3Abin16_01382	BMS3Bbin16_00109, BMS3Bbin16_00399, BMS3Bbin16_00806, BMS3Bbin16_01223	RLG57838, RLG57113, RLG57115, RLG56619, RLG56329, RLG55337, RLG57059, RLG55725	RLG60262, RLG59546, RLG59340
K03389	*hdrB*	IPdc08_00599	BMS3Bbin15_00265, BMS3Bbin15_00340, BMS3Bbin15_01422	2730017249, 2730017156, 2730016549, 2730015752	2730026941, 2730026878, 2730026794, 2730026506, 2730026470	2730022197, 2730021085	BMS3Abin16_00749, BMS3Abin16_00750, BMS3Abin16_01600		RLG55339, RLG56013, RLG55727	
K03390	*hdrC*	IPdc08_00172, IPdc08_00600	BMS3Bbin15_01423	2730017157, 2730016548	2730026940, 2730026877, 2730026795	2730022198, 2730021086	BMS3Abin16_00751, BMS3Abin16_01601		RLG57968, RLG56012	RLG59108
K08264	*hdrD*				2730027667	2730022093, 2730020998				

**Catalase**										
catalase [EC:1.11.1.6]										
K03781	*katE*	IPdc08_01118	BMS3Bbin15_00037	2730016138	2730027132, 2730026172					
**F-type ATPase**										
V/A-type H+/Na+-transporting ATPase subunit A [EC:3.6.3.14 3.6.3.15]										
K02117	*atpA*	IPdc08_01382	BMS3Bbin15_00680		2730027049	2730021980	BMS3Abin16_01267	BMS3Bbin16_00277	RLG56364	RLG58489
K02118	*atpB*	IPdc08_01381	BMS3Bbin15_00681			2730021979	BMS3Abin16_01268	BMS3Bbin16_00278	RLG56363	RLG58490
K02119	*atpC*	IPdc08_00880	BMS3Bbin15_00725	2730017071	2730027002	2730020662	BMS3Abin16_00514, BMS3Abin16_01187		RLG58611	RLG57646
K02120	*atpD*	IPdc08_01380	BMS3Bbin15_00682			2730021978	BMS3Abin16_01269	BMS3Bbin16_00279	RLG56362	RLG58491
K02121	*atpE*	IPdc08_01384	BMS3Bbin15_00678		2730027047	2730020453	BMS3Abin16_00298	BMS3Bbin16_00275	RLG56072	
K02122	*atpG/H*	IPdc08_01383	BMS3Bbin15_00679		2730027048	2730021981	BMS3Abin16_00297	BMS3Bbin16_00276	RLG56365	
K02123	*atpI*	IPdc08_00878	BMS3Bbin15_00727, BMS3Bbin15_01579	2730017341, 2730017073	2730027534, 2730027004, 2730026531	2730021120, 2730020669	BMS3Abin16_00517, BMS3Abin16_01189	BMS3Bbin16_00825	RLG57645	RLG58613, RLG58946
K02124	*atpK*	IPdc08_00879	BMS3Bbin15_00726	2730017072	2730027003	2730020668	BMS3Abin16_00516, BMS3Abin16_01188		RLG57649	RLG58612
**Cytochrome c oxidase**										
cytochrome c oxidase [EC:1.9.3.1]										
K02275	*coxB*			2730016138	2730027132, 2730026172					
K02274	*coxA*			2730016139	2730027133					
K02257	*cox10*			2730016141	2730027134, 2730026219, 2730025957					
**Flagellar system**										
**Archaeal flagellar proteins**										
V/A-type H+/Na+-transporting ATPase subunit A [EC:3.6.3.14 3.6.3.15]										
K07325	*flaB*		BMS3Bbin15_00807	2730015856	2730027806, 2730027790, 2730027789, 2730025471, 2730025470		BMS3Abin16_00549, BMS3Abin16_00550, BMS3Abin16_01002	BMS3Bbin16_01146	RLG55889, RLG57983	RLG60299, RLG60300
K07822	*flaC*		BMS3Bbin15_00808							
K07330	*flaG*		BMS3Bbin15_00812	2730015858, 2730015857	2730027805, 2730027804		BMS3Abin16_00546	BMS3Bbin16_01143	RLG55583, RLG55584, RLG53944, RLG53945	
K07331	*flaH*		BMS3Bbin15_00813		2730026932		BMS3Abin16_00545	BMS3Bbin16_01142	RLG55585	
K07332	*flaI*	IPdc08_00720	BMS3Bbin15_00138, BMS3Bbin15_00814, BMS3Bbin15_01248, BMS3Bbin15_01918	2730017358, 2730017163, 2730017162, 2730017054, 2730016383	2730027961, 2730027653, 2730027558, 2730026978, 2730026934, 2730026933, 2730025549	2730021966, 2730021526, 2730020475	BMS3Abin16_00543, BMS3Abin16_00544, BMS3Abin16_00598, BMS3Abin16_01483, BMS3Abin16_01658	BMS3Bbin16_00304, BMS3Bbin16_00407, BMS3Bbin16_01120	RLG58308, RLG57595, RLG55586, RLG55109, RLG55055, RLG57041, RLG56838, RLG56019, RLG53911	RLG58670, RLG60289, RLG60290, RLG59615, RLG59596, RLG59125.1
K07333	*flaJ*	IPdc08_01202	BMS3Bbin15_00149, BMS3Bbin15_00150, BMS3Bbin15_00815, BMS3Bbin15_01247	2730017357	2730027654, 2730026979, 2730026935	2730021967	BMS3Abin16_00542	BMS3Bbin16_00018, BMS3Bbin16_01121	RLG58307, RLG55056, RLG57042, RLG56839, RLG56840	RLG58668, RLG58669, RLG60291
K07991	*flaK*	IPdc08_00724, IPdc08_01149	BMS3Bbin15_00432, BMS3Bbin15_01915	2730016439, 2730016195	2730027631, 2730025248	2730022738, 2730021405	BMS3Abin16_01613	BMS3Bbin16_00376		RLG58673, RLG59456
**Chemotaxis proteins**										
MCPs										
K03406	*mcp*			2730017409, 2730016523, 2730016507, 2730016203, 2730015944	2730027576, 2730027015, 2730026370, 2730026315, 2730025399, 2730025221	2730021173	BMS3Abin16_00267	BMS3Bbin16_00770		RLG59745
chemotaxis family										
K03407	*cheA*			2730016503, 2730015865	2730027798, 2730025668, 2730025225	2730021169	BMS3Abin16_00270	BMS3Bbin16_00773	RLG56767, RLG56317, RLG56977	
K03408	*cheW*			2730016506	2730026369, 2730025222	2730021172	BMS3Abin16_00268	BMS3Bbin16_00771		RLG59749
K03410	*cheC*			2730015864	2730027799, 2730025667, 2730025226	2730021168	BMS3Abin16_00271, BMS3Abin16_00274, BMS3Abin16_00275	BMS3Bbin16_00774	RLG56765, RLG56766, RLG56975, RLG56976	
K03411	*cheD*			2730015863	2730027800, 2730025666, 2730025227	2730021167	BMS3Abin16_00272	BMS3Bbin16_00775	RLG56764, RLG56974	
K03412	*cheB*			2730016608, 2730016587, 2730016504	2730027403, 2730026847, 2730025224	2730021170	BMS3Abin16_00269	BMS3Bbin16_00772	RLG56319	RLG59748
K03413	*cheY*			2730016505, 2730015866	2730027797, 2730025669, 2730025223	2730021171	BMS3Abin16_00266	BMS3Bbin16_00769	RLG56318	RLG59747
K00575	*cheR*			2730016609, 2730016588	2730027402, 2730026848, 2730025228	2730021723	BMS3Abin16_00273		RLG57988	
**Transporter**										
**ABC Transporters**										
**Mineral and organic ion transporters**										
Molybdate transporter [MD:M00189]										
K02020	*modA*	IPdc08_01479	BMS3Bbin15_01929	2730016781	2730025260		BMS3Abin16_00470	BMS3Bbin16_00742		
K02018	*modB*	IPdc08_01478	BMS3Bbin15_01928	2730016782	2730025261		BMS3Abin16_00469	BMS3Bbin16_00741		
K02017	*modC*	IPdc08_00490	BMS3Bbin15_01853, BMS3Bbin15_01927	2730016783	2730025262					
Molybdate/tungstate transporter [MD:M00423]										
K15495	*wtpA*	IPdc08_01642	BMS3Bbin15_00222						RLG55445, RLG55887	
K15496	*wtpB*	IPdc08_01644	BMS3Bbin15_00224						RLG55444, RLG55886	
K15497	*wtpC*		BMS3Bbin15_00223						RLG55443	RLG59329
Tungstate transporter [MD:M00186]										
K05772	*tupA*		BMS3Bbin15_00225		2730027097, 2730026423	2730021477	BMS3Abin16_00114			
K05773	*tupB*		BMS3Bbin15_00226	2730015975	2730026424	2730021476	BMS3Abin16_00113			

K06857	*tupC*		BMS3Bbin15_00227		2730026425	2730021475	BMS3Abin16_00112			
NitT/TauT family transporter [MD:M00188]										
K02051	*tauA*			2730016926	2730027138	2730021752, 2730021003, 2730020982			RLG58135, RLG57023	
K02050	*tauC*					2730021751, 2730020991, 2730020990			RLG58137	
K02049	*tauB*					2730022561, 2730020993, 2730020992			RLG58136, RLG57020	
**Saccharide, polyol, and lipid transporters**										
Putative multiple sugar transporter [MD:M00207]										
K02027	*ugpB*	IPdc08_01344								
K02025	*ugpA*	IPdc08_01343								
K02026	*ugpE*	IPdc08_01342								
Putative ABC transporter [MD:M00211]										
K02069	-		BMS3Bbin15_01726							
K02068	-		BMS3Bbin15_01725							
Phosphate transporter [MD:M00222]										
K02040	*pstS*	IPdc08_01395	BMS3Bbin15_01022			2730020730	BMS3Abin16_01251	BMS3Bbin16_00335	RLG56413	
K02037	*pstC*	IPdc08_01394	BMS3Bbin15_01023		2730026351	2730020731	BMS3Abin16_01252	BMS3Bbin16_00336	RLG56415	
K02038	*pstA*	IPdc08_01393	BMS3Bbin15_01024		2730026352	2730020732	BMS3Abin16_01253	BMS3Bbin16_00337		
K02036	*pstB*	IPdc08_01392	BMS3Bbin15_01025		2730026353	2730020733	BMS3Abin16_01254	BMS3Bbin16_00338		
Branched-chain amino acid transporter [MD:M00237]										
K01999	*livK*			2730016623, 2730016308	2730027364, 2730026038				RLG58501, RLG55859	RLG59790, RLG58538
K01997	*livH*			2730017033, 2730016615, 2730016430	2730026039				RLG58499	RLG58536
K01998	*livM*			2730017034, 2730016624, 2730016614, 2730016431					RLG58498, RLG55857	RLG58537
K01995	*livG*			2730016623, 2730016308	2730026037				RLG58501, RLG55859	RLG59790, RLG58538
K01996	*livF*			2730016309					RLG58502, RLG54307	RLG59793
Neutral amino acid transporter [MD:M00322]										
K11956	*natD*			2730016625						
**Peptide and nickel transporters**										
Oligopeptide transporter [MD:M00439]										
K10823	*oppF*	IPdc08_00146				2730021956				
Peptides/nickel transporter [MD:M00239]										
K02035	*ddpA*	IPdc08_00142	BMS3Bbin15_01735			2730021960			RLG57282	RLG58757
K02033	*dppB*	IPdc08_00143	BMS3Bbin15_01734			2730021959			RLG57279, RLG56049	RLG58753
K02034	*dppC*	IPdc08_00144	BMS3Bbin15_01733			2730021958			RLG57280, RLG56048	RLG58754
K02031	*gsiA*	IPdc08_00145				2730021957			RLG57281, RLG56047	RLG58755
**Metallic cation, iron-siderophore and vitamin B12 transporters**										
Iron complex transporter [MD:M00240]										
K02016	*fepB*	IPdc08_01179	BMS3Bbin15_00418				BMS3Abin16_00231			
K02015	*fepD*		BMS3Bbin15_00417				BMS3Abin16_01038	BMS3Bbin16_01155		
K02013	*fepC*		BMS3Bbin15_00416				BMS3Abin16_01039	BMS3Bbin16_01154		
Zinc transporter [MD:M00242]										
K09816	*znuB*	IPdc08_00762	BMS3Bbin15_01722							
K09817	*znuC*	IPdc08_00761	BMS3Bbin15_01723							
Cobalt transporter [MD:M00245]/Nickel transporter [MD:M00246]										
K02006	*cbiO*		BMS3Bbin15_01199, BMS3Bbin15_01643	2730017360		2730021325, 2730022234	BMS3Abin16_00245			
K02008	*cbiQ*		BMS3Bbin15_01200, BMS3Bbin15_01642			2730021324, 2730022235	BMS3Abin16_00246	BMS3Bbin16_01202		
K02009	*cbiN*		BMS3Bbin15_01641			2730021326, 2730022236	BMS3Abin16_00247	BMS3Bbin16_01201		
K02007	*cbiM*		BMS3Bbin15_01203, BMS3Bbin15_01640			2730021327, 2730022237	BMS3Abin16_00248	BMS3Bbin16_01200		
Biotin transporter [MD:M00581]/Energy-coupling factor transporter [MD:M00582]										
K03523	*bioY*	IPdc08_01836	BMS3Bbin15_01338			2730020758	BMS3Abin16_00764, BMS3Abin16_01670	BMS3Bbin16_00116	RLG58497	
**ABC-2 type and other transporters**										
Copper-processing transporter [MD:M00762]										
K19341	*nosY*			2730016753	2730026182					
K19340	*nosF*			2730016752	2730026181					
Heme transporter [MD:M00259]										
K02195	*ccmC*		BMS3Bbin15_01808	2730016514, 2730016109	2730027661, 2730027173, 2730026229	2730022454	BMS3Abin16_01616			RLG59655
K02194	*ccmB*		BMS3Bbin15_01809	2730017186	2730027660	2730022455	BMS3Abin16_01617			RLG59654
Putative ABC transporter [MD:M00258]										
K02004	-	IPdc08_00260, IPdc08_00261, IPdc08_00506, IPdc08_00590	BMS3Bbin15_00441, BMS3Bbin15_01566, BMS3Bbin15_01599, BMS3Bbin15_01600	2730017333, 2730017298, 2730017197, 2730017196, 2730016991, 2730016990, 2730016658	2730027197, 2730027196, 2730026863, 2730026102, 2730025916, 2730025806, 2730025804	2730021719, 2730021506, 2730021505	BMS3Abin16_01281, BMS3Abin16_01282, BMS3Abin16_01539, BMS3Abin16_01540	BMS3Bbin16_00761	RLG58551, RLG57160, RLG58161	
K02003	-	IPdc08_00504, IPdc08_00588, IPdc08_01103	BMS3Bbin15_00049, BMS3Bbin15_00442, BMS3Bbin15_01238	2730017334, 2730017302, 2730017297, 2730016655	2730026864, 2730026859, 2730026440, 2730026103, 2730026098, 2730025805, 2730025624	2730021507	BMS3Abin16_01283, BMS3Abin16_01541	BMS3Bbin16_00619, BMS3Bbin16_00759	RLG58553, RLG57158, RLG57159, RLG58162, RLG58163	
ABC-2 type transporter [MD:M00254]										
K01992	-	IPdc08_00593, IPdc08_00677, IPdc08_00771	BMS3Bbin15_00325, BMS3Bbin15_00345, BMS3Bbin15_00654	2730017445, 2730016469, 2730016270	2730026596	2730021778, 2730021768, 2730021710, 2730021709, 2730021485, 2730020780	BMS3Abin16_01209		RLG56996, RLG56432, RLG56921	RLG60105, RLG59791, RLG59653

K01990	-	IPdc08_00435, IPdc08_00592, IPdc08_00678, IPdc08_00770, IPdc08_01387	BMS3Bbin15_00324, BMS3Bbin15_00346, BMS3Bbin15_00653, BMS3Bbin15_01893	2730017444, 2730017389, 2730017185, 2730016470, 2730016271, 2730016242	2730027890, 2730027824, 2730027103, 2730026998, 2730026899, 2730026595, 2730026253	2730022456, 2730022356, 2730021779, 2730021767, 2730021711, 2730020781	BMS3Abin16_01208, BMS3Abin16_01618		RLG56433, RLG57215, RLG56922, RLG54736	RLG60106
**Solute Carrier Family (SLC)**										
Na+-sulfate/carboxylate cotransporter										
K14445	*citT*			2730016520			BMS3Abin16_00625	BMS3Bbin16_01183	RLG55239	
**Major Facilitator Superfamily (MFS)**										
**Nitrate/nitrite transporters**										
Nitrate/nitrite porter (NNP) family [TC:2.A.1.8]										
K02575	*narK*				2730025438					
**Other Transporters**										
**Pores ion channels [TC:1]**										
voltage-gated potassium channel										
K10716	*kch*	IPdc08_00619, IPdc08_00620	BMS3Bbin15_01672	2730016361, 2730016360	2730027630, 2730027629, 2730025247, 2730025246	2730021570, 2730021569	BMS3Abin16_00599	BMS3Bbin16_00406		
ammonium transporter, Amt family										
K03320	*amt*	IPdc08_00832	BMS3Bbin15_00197	2730016914	2730027813, 2730026360		BMS3Abin16_00086	BMS3Bbin16_00071		
Ca-activated chloride channel homolog										
K07114	*yfbK*					2730020666				
magnesium transporter										
K06213	*mgtE*	IPdc08_00587	BMS3Bbin15_00348	2730015987	2730027497, 2730026025	2730021652	BMS3Abin16_00747, BMS3Abin16_01775	BMS3Bbin16_00735	RLG58097	RLG58638
K03284	*corA*						BMS3Abin16_00868, BMS3Abin16_00869	BMS3Bbin16_00481		
fluoride exporter										
K06199	*crcB*							BMS3Bbin16_00396		
**Electrochemical potential-driven transporters [TC:2]**										
cobalt-zinc-cadmium efflux system protein										
K16264	*czcD*			2730017013	2730026280, 2730025883	2730021539				
zinc and cadmium transporter										
K16267	*zipB*				2730027696	2730022549	BMS3Abin16_00063			
drug/metabolite transporter, DME family										
K03298	*rhaT*					2730022522				
small multidrug resistance family-3 protein										
K09771	*ynfA*		BMS3Bbin15_00698				BMS3Abin16_00932			
cation:H+ antiporter										
K07301	*yrbG*	IPdc08_01879	BMS3Bbin15_00426	2730017031, 2730016576, 2730015925	2730027128, 2730026728, 2730026713, 2730026122	2730021927, 2730020513	BMS3Abin16_01854	BMS3Bbin16_00219		RLG60074
trk system potassium uptake protein										
K03498	*trkH*		BMS3Bbin15_00573	2730016303, 2730016647, 2730017105	2730026545, 2730026663	2730021518	BMS3Abin16_00578, BMS3Abin16_00590, BMS3Abin16_00591	BMS3Bbin16_00408, BMS3Bbin16_00409, BMS3Bbin16_00654	RLG55546, RLG57603	
K03499	*trkA*		BMS3Bbin15_01704	2730016302	2730026546, 2730026664	2730020455	BMS3Abin16_01361	BMS3Bbin16_00003	RLG56074, RLG54206	
high-affinity nickel-transport protein										
K07241	*nixA*	IPdc08_01180, IPdc08_01785	BMS3Bbin15_00419, BMS3Bbin15_01380	2730017114, 2730016021	2730027324, 2730025341	2730022088				
sulfate permease, SulP family										
K03321		IPdc08_00413	BMS3Bbin15_01111		2730027603, 2730027093	2730021933, 2730021301				
arsenite transporter										
K03325	*arsB*	IPdc08_00769	BMS3Bbin15_00652				BMS3Abin16_01344, BMS3Abin16_01411	BMS3Bbin16_00744, BMS3Bbin16_01109		
multicomponent Na+:H+ antiporter										
K05566	*mnhB*					2730022252, 2730022251	BMS3Abin16_01192			
K05567	*mnhC*					2730022250	BMS3Abin16_01191			
K05568	*mnhD*					2730022249, 2730022248, 2730022246	BMS3Abin16_00096, BMS3Abin16_00334, BMS3Abin16_01190, BMS3Abin16_01693	BMS3Bbin16_00292, BMS3Bbin16_00732		
K05569	*mnhE*					2730022256	BMS3Abin16_01197			
K05570	*mnhF*					2730022255	BMS3Abin16_01196			
K05571	*mnhG*					2730022254	BMS3Abin16_01195			
multiple antibiotic resistance protein										
K05595	*marC*	IPdc08_01750	BMS3Bbin15_01348	2730016721		2730020723	BMS3Abin16_01803	BMS3Bbin16_00705		
high-affinity iron transporter										
K07243	*ftr1*			2730016508		2730021972	BMS3Abin16_01585	BMS3Bbin16_00303		
TolB protein										
K03641	*tolB*						BMS3Abin16_00455, BMS3Abin16_01467	BMS3Bbin16_00526		
**A-type ATPase**										
arsenite/tail-anchored protein-transporting ATPase [EC:3.6.3.16 3.6.3.-]										
K01551	*arsA*	IPdc08_00765	BMS3Bbin15_00647, BMS3Bbin15_01485		2730026993	2730021189	BMS3Abin16_01389	BMS3Bbin16_01269		
**P-type ATPase**										
Cd2+/Zn2+-exporting ATPase [EC:3.6.3.3 3.6.3.5]										
K01534	*cadA*	IPdc08_00564	BMS3Bbin15_01753			2730020799	BMS3Abin16_01806	BMS3Bbin16_01028		
Cu2+-exporting ATPase [EC:3.6.3.4 3.6.3.54]										
K17686	*copA*	IPdc08_01158	BMS3Bbin15_01062	2730016742, 2730016199	2730027035, 2730025729	2730021923, 2730021840, 2730021179				
K01533	*copB*	IPdc08_01536	BMS3Bbin15_01718	2730015957	2730027144, 2730027143		BMS3Abin16_01294	BMS3Bbin16_01033		
**Others**										
Sulfite exporter TauE/SafE family (TSUP)										
K07090	*TauE*	IPdc08_00236, IPdc08_00239	BMS3Bbin15_01035, BMS3Bbin15_01170, BMS3Bbin15_01520	2730017361, 2730016645	2730027347, 2730025896, 2730025602	2730022475, 2730020925, 2730020691	BMS3Abin16_00464	BMS3Bbin16_00736	RLG55060, RLG57321	RLG59871
ferrous iron transport protein B										
K04759	*feoB*			2730017023	2730027045	2730021973	BMS3Abin16_01583	BMS3Bbin16_00301	RLG54959	RLG59901, RLG59046

Table S2. List of CDSs for c-type cytochromes encoded in the MAGs of Ca. Hydrothermarchaeota

Gene locus/ID	Number of CXXCH motifs	Number of CXXXCH motifs	total	Predicted subcellular localization	Psortb score
**MBGE-A**					
BMS3Bbin15 (5 MHCs in total 16 Cyc)					
BMS3Bbin15_00096	1	0	1	Cytoplasmic Membrane	9.82
BMS3Bbin15_00340	1	0	1	Cytoplasmic	7.5
BMS3Bbin15_00614	1	0	1	Cytoplasmic	7.5
BMS3Bbin15_00626	1	0	1	Cytoplasmic Membrane	9.75
BMS3Bbin15_00628	1	0	1	Unknown	
BMS3Bbin15_00819	5	0	5	Unknown	
BMS3Bbin15_00831	1	0	1	Cytoplasmic	7.5
BMS3Bbin15_01005	1	0	1	Cytoplasmic	7.5
BMS3Bbin15_01010	1	0	1	Cytoplasmic	7.5
BMS3Bbin15_01030	1	0	1	Cytoplasmic Membrane	10
BMS3Bbin15_01038	1	1	2	Unknown	
BMS3Bbin15_01092	5	0	5	Unknown	
BMS3Bbin15_01490	1	0	1	Cytoplasmic	7.5
BMS3Bbin15_01570	1	0	1	Cytoplasmic	9.96
BMS3Bbin15_01689	1	0	1	Cytoplasmic	9.96
BMS3Bbin15_01759	3	0	3	Unknown	
BMS3Bbin15_01769	1	0	1	Cytoplasmic	9.96
BMS3Bbin15_01896	3	0	3	Unknown	
BMS3Bbin15_01907	1	0	1	Cytoplasmic	7.5
IPdc08 (no MHC in total 10 Cyc)					
IPdc08_00099	1	0	1	Cytoplasmic	7.5
IPdc08_00126	1	0	1	Cytoplasmic	7.5
IPdc08_00599	1	0	1	Cytoplasmic	7.5
IPdc08_00672	1	0	1	Unknown	
IPdc08_00705	1	0	1	Unknown	
IPdc08_00860	1	0	1	Cytoplasmic	7.5
IPdc08_00990	1	0	1	Cytoplasmic	9.96
IPdc08_01280	1	0	1	Cytoplasmic	7.5
IPdc08_01473	1	0	1	Cytoplasmic	7.5
IPdc08_01597	1	0	1	Cytoplasmic	7.5
**MBGE-B**					
JdFR-16 (24 MHCs in total 34 Cyc)					
2730015732	25	0	25	Extracellular	6.8
2730015733	3	0	3	Cytoplasmic	7.5
2730015734	4	0	4	Unknown	
2730015736	7	0	7	Unknown	
2730015739	5	0	5	Cytoplasmic	7.5
2730015912	1	0	1	Cytoplasmic	7.5
2730015968	1	0	1	Cytoplasmic	7.5
2730016221	2	0	2	Unknown	
2730016224	1	0	1	Cytoplasmic Membrane	10
2730016245	5	0	5	Cytoplasmic	7.5
2730016285	1	0	1	Cytoplasmic	7.5
2730016458	1	0	1	Cytoplasmic	7.5
2730016478	3	0	3	Cytoplasmic	7.5
2730016494	8	0	8	Unknown	
2730016549	1	0	1	Cytoplasmic	7.5
2730016595	1	0	1	Cytoplasmic Membrane	9.82

2730016654	2	0	2	Cytoplasmic	7.5
2730016774	1	0	1	Cytoplasmic Membrane	9.82
2730016818	8	0	8	Extracellular	6.8
2730016885	5	0	5	Cytoplasmic	7.5
2730016946	17	1	18	Extracellular	8.82
2730016947	27	0	27	Cellwall/Extracellular	
2730016948	4	0	4	Cytoplasmic	7.5
2730016949	4	0	4	Unknown	
2730016952	6	1	7	Unknown	
2730016954	5	0	5	Cytoplasmic	7.5
2730016958	4	0	4	Cytoplasmic	7.5
2730016969	14	0	14	Cytoplasmic	7.5
2730017029	1	0	1	Cytoplasmic Membrane	9.82
2730017117	3	0	3	Cytoplasmic	7.5
2730017175	1	0	1	Cytoplasmic	7.5
2730017282	2	0	2	Unknown	
2730017363	9	0	9	Unknown	
2730017414	4	1	5	Cytoplasmic	7.5
JdFR-17 (31 MHCs in total 49 Cyc)					
2730025241	7	0	7	Extracellular	6.8
2730025253	1	0	1	Cytoplasmic Membrane	9.82
2730025344	3	0	3	Cytoplasmic	7.5
2730025433	2	0	2	Cytoplasmic	7.5
2730025435	1	0	1	Cytoplasmic Membrane	9.82
2730025452	5	0	5	Cytoplasmic	7.5
2730025455	4	0	4	Unknown	
2730025456	4	0	4	Cytoplasmic	7.5
2730025457	34	0	34	Extracellular	6.8
2730025478	1	0	1	Unknown	
2730025488	3	1	4	Cytoplasmic	7.5
2730025514	8	0	8	Cytoplasmic	7.5
2730025573	8	0	8	Unknown	
2730025696	1	0	1	Cytoplasmic	7.5
2730025757	14	0	14	Cytoplasmic	7.5
2730025825	8	0	8	Cytoplasmic	7.5
2730025915	1	0	1	Cytoplasmic	7.5
2730026035	1	0	1	Cytoplasmic	7.5
2730026092	4	1	5	Cytoplasmic	7.5
2730026136	1	0	1	Cytoplasmic Membrane	9.82
2730026139	8	0	8	Cytoplasmic	7.5
2730026295	5	0	5	Unknown	
2730026326	1	1	2	Cytoplasmic	7.5
2730026367	1	0	1	Cytoplasmic	7.5
2730026434	4	1	5	Cytoplasmic	7.5
2730026519	5	0	5	Cytoplasmic	7.5
2730026522	7	0	7	Unknown	
2730026524	4	0	4	Unknown	
2730026525	3	0	3	Cytoplasmic	7.5
2730026526	25	0	25	Extracellular	6.8
2730026602	8	0	8	Unknown	
2730026736	5	0	5	Cytoplasmic	7.5
2730026794	1	0	1	Cytoplasmic	7.5
2730026878	1	0	1	Cytoplasmic	7.5

2730026890	8	0	8	Unknown	
2730026895	5	0	5	Cytoplasmic	7.5
2730026994	5	0	5	Cytoplasmic	7.5
2730027135	1	0	1	Cytoplasmic Membrane	9.82
2730027154	1	0	1	Cytoplasmic	7.5
2730027267	1	0	1	Cytoplasmic	7.5
2730027355	1	0	1	Cytoplasmic Membrane	9.82
2730027426	8	0	8	Cytoplasmic	7.5
2730027441	1	0	1	Cytoplasmic	7.5
2730027551	1	0	1	Cytoplasmic	7.5
2730027687	7	0	7	Cytoplasmic	7.5
2730027690	1	0	1	Cytoplasmic Membrane	10
2730027761	1	0	1	Cytoplasmic	7.5
2730027784	8	0	8	Cytoplasmic	7.5
2730027827	5	0	5	Cytoplasmic	7.5
**MBGE-C**					
JdFR-18 (21 MHCs in total 40 Cyc)					
2730020450	1	0	1	Cytoplasmic	7.5
2730020592	2	0	2	Cytoplasmic Membrane	9.82
2730020595	1	0	1	Cytoplasmic	7.5
2730020707	1	0	1	Cytoplasmic	7.5
2730020743	1	0	1	Cytoplasmic Membrane	9.82
2730020858	1	0	1	Unknown	
2730020935	2	0	2	Cytoplasmic	7.5
2730020966	3	0	3	Unknown	
2730021000	1	0	1	Cytoplasmic	7.5
2730021043	7	0	7	Extracellular	8.82
2730021044	2	0	2	Unknown	
2730021085	1	0	1	Cytoplasmic	7.5
2730021094	9	0	9	Cytoplasmic	7.5
2730021143	8	0	8	Unknown	
2730021145	1	0	1	Cytoplasmic Membrane	9.82
2730021152	6	0	6	Cytoplasmic	7.5
2730021159	1	0	1	Cytoplasmic	7.5
2730021181	1	0	1	Unknown	
2730021188	4	1	5	Cytoplasmic Membrane	9.26
2730021438	5	0	5	Cytoplasmic	7.5
2730021716	10	0	10	Cytoplasmic Membrane	9.06
2730021718	1	0	1	Cytoplasmic	7.5
2730021849	2	0	2	Cytoplasmic	7.5
2730021875	1	0	1	Cytoplasmic	7.5
2730021878	1	0	1	Cytoplasmic	7.5
2730021932	1	0	1	Cytoplasmic	7.5
2730021943	1	0	1	Cytoplasmic	7.5
2730022042	5	0	5	Cytoplasmic	7.5
2730022071	67	0	67	Cellwall	9.4
2730022073	4	0	4	Unknown	
2730022074	10	0	10	Cytoplasmic Membrane	9.31
2730022352	4	1	5	Cytoplasmic	7.5
2730022364	1	0	1	Cytoplasmic	7.5
2730022375	1	0	1	Cytoplasmic	7.5
2730022399	1	0	1	Cytoplasmic	7.5
2730022441	6	0	6	Cytoplasmic	7.5

2730022444	3	0	3	Unknown	
2730022445	3	0	3	Extracellular	6.8
2730022446	5	0	5	Cytoplasmic	7.5
2730022501	1	0	1	Cytoplasmic	9.96
**MBGE-D**					
BMS3Abin16 (13 MHCs in total 34 Cyc)					
BMS3Abin16_00159	1	0	1	Cytoplasmic	9.96
BMS3Abin16_00405	1	0	1	Cytoplasmic	8.61
BMS3Abin16_00429	3	0	3	Unknown	
BMS3Abin16_00431	5	0	5	Cytoplasmic	7.5
BMS3Abin16_00433	5	0	5	Cytoplasmic	7.5
BMS3Abin16_00434	5	0	5	Cytoplasmic	7.5
BMS3Abin16_00450	1	0	1	Cytoplasmic	7.5
BMS3Abin16_00497	3	0	3	Cytoplasmic	7.5
BMS3Abin16_00531	1	0	1	Cytoplasmic	7.5
BMS3Abin16_00749	1	0	1	Cytoplasmic	7.5
BMS3Abin16_00750	1	0	1	Cytoplasmic	7.5
BMS3Abin16_00829	1	0	1	Cytoplasmic Membrane	9.82
BMS3Abin16_00851	1	0	1	Cytoplasmic	7.5
BMS3Abin16_00872	1	0	1	Cytoplasmic	7.5
BMS3Abin16_01022	2	0	2	Unknown	
BMS3Abin16_01077	1	0	1	Cytoplasmic	7.5
BMS3Abin16_01142	2	0	2	Unknown	
BMS3Abin16_01223	1	0	1	Cytoplasmic	7.5
BMS3Abin16_01270	4	0	4	Unknown	
BMS3Abin16_01275	1	0	1	Cytoplasmic Membrane	9.82
BMS3Abin16_01391	1	0	1	Cytoplasmic Membrane	9.82
BMS3Abin16_01423	1	0	1	Cytoplasmic	7.5
BMS3Abin16_01495	3	0	3	Unknown	
BMS3Abin16_01600	1	0	1	Cytoplasmic	7.5
BMS3Abin16_01629	1	0	1	Cytoplasmic Membrane	9.75
BMS3Abin16_01631	1	0	1	Cytoplasmic Membrane	9.75
BMS3Abin16_01633	5	0	5	Unknown	
BMS3Abin16_01637	5	0	5	Unknown	
BMS3Abin16_01638	5	0	5	Cytoplasmic	7.5
BMS3Abin16_01639	1	0	1	Unknown	
BMS3Abin16_01707	2	0	2	Unknown	
BMS3Abin16_01726	1	0	1	Cytoplasmic	7.5
BMS3Abin16_01738	1	0	1	Unknown	
BMS3Abin16_01842	1	0	1	Cytoplasmic	7.5
BMS3Bbin16 (7 MHCs in total 19 Cyc)					
BMS3Bbin16_00187	5	0	5	Unknown	
BMS3Bbin16_00208	1	0	1	Cytoplasmic	7.5
BMS3Bbin16_00249	1	0	1	Cytoplasmic	9.96
BMS3Bbin16_00280	4	0	4	Unknown	
BMS3Bbin16_00372	1	0	1	Cytoplasmic	7.5
BMS3Bbin16_00395	1	0	1	Cytoplasmic	7.5
BMS3Bbin16_00609	5	0	5	Unknown	
BMS3Bbin16_00611	1	0	1	Cytoplasmic Membrane	9.75
BMS3Bbin16_00612	1	0	1	Cytoplasmic Membrane	9.75
BMS3Bbin16_00805	4	0	4	Cytoplasmic	7.5
BMS3Bbin16_00814	5	0	5	Cytoplasmic	7.5
BMS3Bbin16_00815	5	0	5	Cytoplasmic	7.5

BMS3Bbin16_00907	1	0	1	Unknown	
BMS3Bbin16_00930	1	0	1	Cytoplasmic	7.5
BMS3Bbin16_01193	5	0	5	Unknown	
BMS3Bbin16_01223	1	0	1	Cytoplasmic	7.5
BMS3Bbin16_01265	1	0	1	Cytoplasmic Membrane	9.82
BMS3Bbin16_01271	1	0	1	Cytoplasmic Membrane	9.82
BMS3Bbin16_01279	1	0	1	Cytoplasmic	7.5
**MBGE-E**					
B51_G15 (2 MHCs in total 11 Cyc)					
RLG55404	1	0	1	CytoplasmicMembrane	9.82
RLG55912	1	0	1	Cytoplasmic	7.5
RLG56299	1	0	1	Cytoplasmic	10
RLG56324	1	0	1	Cytoplasmic	7.5
RLG56988	1	0	1	CytoplasmicMembrane	9.82
RLG57147	1	0	1	Cytoplasmic	7.5
RLG57967	1	0	1	Cytoplasmic	7.5
RLG58210	10	0	10	Cytoplasmic	7.5
RLG58216	1	0	1	Cytoplasmic	7.5
RLG58354	9	0	9	Cytoplasmic	7.5
RLG58698	1	0	1	Cytoplasmic	7.5
B60_G1 (9 MHCs in total 22 Cyc)					
RLG54237	1	0	1	Cytoplasmic	9.96
RLG54460	0	1	1	Cytoplasmic	7.5
RLG54519	1	0	1	Cytoplasmic	9.96
RLG55163	7	0	7	Cytoplasmic	7.5
RLG55548	0	1	1	Cytoplasmic	7.5
RLG55608	1	0	1	CytoplasmicMembrane	9.82
RLG56218	5	0	5	Cytoplasmic	7.5
RLG56348	5	0	5	Extracellular	8.82
RLG56621	4	0	4	Unknown	
RLG56690	1	0	1	Unknown	
RLG56768	1	0	1	Unknown	
RLG57689	5	0	5	Unknown	
RLG58772	1	0	1	Extracellular	8.82
RLG59084	1	0	1	Cytoplasmic	7.5
RLG59107	1	0	1	CytoplasmicMembrane	9.82
RLG59167	3	0	3	CytoplasmicMembrane	9.82
RLG59168	0	1	1	CytoplasmicMembrane	9.82
RLG59630	1	0	1	CytoplasmicMembrane	9.82
RLG59728	4	0	4	Cytoplasmic	7.5
RLG59729	7	0	7	Cytoplasmic	7.5
RLG60207	1	1	2	Cytoplasmic	9.96
RLG60264	1	0	1	Cytoplasmic	7.5

Table S3. List of the locations where 16S rRNA genes of Ca. Hydrothermarchaeota were detected (Silva database)

Location	Isolation source	Latitude	Longitude	Clone library name	% MBGE clones of total archaeal clones	% MBGE clones of total prokaryotic clones	% archaeal cells of total prokaryotic cells	% MBGE cells of total prokaryotic cells	Accession number of representative clone	Reference (pubmed ID or DOI)
Myojin Knoll, Izu-Ogasawara arc	clear smoker chimney	32.1034667N	139.8645446E	MC1	22.0%	N.D.	17.2%	3.8%	AB019754	10430559
NW Atlantic Ocean	deep-sea sediment	39.332N	70.665522W	CRA	N.D.	N.D.	N.D.	N.D.	AF119124	10508063
NW Atlantic Ocean	deep-sea sediment	37.3871667N	68.8346887W	APA	N.D.	N.D.	N.D.	N.D.	AF119139	
Gulf of Papua, Southeastern Papua New Guinea	estuarine sediments	8.099S	144.8048113E	TG85	N.D.	1.1%	N.D.	N.D.	AF193559	10967214
Douro River estuary, Portugal	intertidal bank	41.1333333N	8.6688554W	-	N.D.	N.D.	N.D.	N.D.	AF201361	11348430
Mothra Vent Field on the Juan de Fuca Ridge	black smoker chimney	48.9166667N	129.1021887W	FZ1aA	52.1%	N.D.	33.0%	17.2%	AY165965	12788766
		48.9166667N	129.1021887W	FZ2bA	31.9%	N.D.	65.0%	20.8%	AY165974	
		48.9166667N	129.1021887W	FZ2a	31.4%	N.D.	65.0%	20.4%	AY165996	
		48.9166667N	129.1021887W	FZ3a	10.0%	N.D.	63.0%	6.3%	AY182126	
		48.9166667N	129.1021887W	FZ3b	8.7%	N.D.	63.0%	5.5%	AY182130	
Iheya North, Mid-Okinawa Trough	inactive hydrothermal sulfide chimney	27.7913333N	126.8941446E	IheA1	35.5%	N.D.	3.10%	1.1%	AB099934	14749907
		27.7913333N	126.8941446E	IheA3	90.0%	N.D.	4.20%	3.8%		
		27.7913333N	126.8941446E	IheA4	83.9%	N.D.	1.80%	1.5%		
Kairei field, Central Indian Ridge		27.7913333N	126.8941446E	IndA1	62.2%	N.D.	0.30%	0.2%		
		27.7913333N	126.8941446E	IndA2	67.9%	N.D.	0.80%	0.5%		
		27.7913333N	126.8941446E	IndA3	85.1%	N.D.	2.20%	1.9%		
		27.7913333N	126.8941446E	IndA4	80.5%	N.D.	3.70%	3.0%		
Rainbow vent field on the Mid-Atlantic Ridge	hydrothermal sediment	36.2291667N	33.9021887W	IR3-2	84.8%	N.D.	N.D.	N.D.	AY354112	15819852
Iheya North, Mid-Okinawa Trough	deep-sea hydrothermal vent chimney	27.7866667N	126.8961446E	-	N.D.	N.D.	N.D.	N.D.	AB175603	16329980
Baby Bare Seamount, the Juan de Fuca Ridge	crustal fluids	47.75N	127.7521887W	FS266	2.5%	N.D.	1.00%	0.0%	AY704378	16343325
Eastern Flank of Juan de Fuca Ridge	black rust formation	47.7666667N	127.7688554W	CORK	2.6%	N.D.	0.04	0.1%	AB260057	17021232
Dongzhaigang National Nature Reserve, Hainan Island	mangrove soil	19.955N	110.5788391E	MKCS	0.8%	N.D.	N.D.	N.D.	DQ363847	17082752
Marker 19 on the East Pacific Rise	deep sea hydrothermal chimeny	36.216667N	33.900000W	-	N.D.	N.D.	N.D.	N.D.	DQ409071	17221162
Kazan mud volcano, Eastern Mediterranean	mud volcano sediment	35.4316667N	30.559478E	Kazan-1	2.1%	N.D.	N.D.	N.D.	AY591977	17431711
Kazan mud volcano, Eastern Mediterranean	mud volcano sediments	35.4316667N	30.559478E	Kazan-2	6.5%	N.D.	N.D.	N.D.	AY591982	
Urania brine lake, Eastern Mediterranean	marine sediment	35.245N	21.479478E	Urania-2	9.1%	N.D.	N.D.	N.D.	AY627483	18422633
Kaiko site, Southern Mariana Trough	iron-rich hydrothermal deposit	13.0987333N	143.6865113E	YS16	73.8%	N.D.	18.0%	13.3%	AB329756	19397679
Snail site, Southern Mariana Trough		12.9527167N	143.617128E	YS18	21.2%	N.D.	12.0%	2.5%	AB329803	
Timor Sea of Australia	hydrocarbon seep sediment	13.654517S	124.711683E	A1	5.5%	N.D.	N.D.	N.D.	FJ175653	19573197
		13.654517S	124.711683E	A5	2.0%	N.D.	N.D.	N.D.	FJ175654	
New Caledonia Basin	subsurface marine sediment	23.12S	163.27E	DNA22T450	N.D.	N.D.	N.D.	N.D.	AM989380	19624712
Snail site, Southern Mariana Trough	crustal fluids	12.9527778N	143.6178113E	Fapm1aA24	2.0%	N.D.	46.2%	0.9%	AB213060	19691504
	hydrothermal vent fluid	12.9527778N	143.6178113E	Fnv	23.5%	N.D.	15.6%	3.7%	AB213067	
Pika site, Southern Mariana Trough	crustal fluids	12.9527778N	143.6178113E	Pamp	20.0%	N.D.	58.1%	11.6%	AB213087	
California hypersaline lake, the Salton Sea	anoxic hypersaline sediment	33.4166667N	115.9188554W	A (0-1)	1.1%	N.D.	99.0%	1.0%	EU329834	19948847
Yonaguni Knoll IV, Southern Okinawa Trough	hydrothermal sediment	24.85N	122.8728113E	763MY-0-5	2.9%	N.D.	10.0%	0.3%	AB301985	20023079
		24.85N	122.8728113E	763MY-15-20	19.4%	N.D.	10.0%	1.9%	AB301993	
Western Pacific	marine sediment	14.8103333N	123.487978E	TWP2	7.9%	N.D.	N.D.	N.D.	GQ410814	20024655
		14.8103333N	123.487978E	TWP3	3.4%	N.D.	N.D.	N.D.	GQ410822	
		14.8103333N	123.487978E	TWP6	3.4%	N.D.	N.D.	N.D.	GQ410832	
Mississippi canyon 118, northern slope of Gulf of Mexico	marine sediment	28.8578333N	88.4941887W	Outside	N.D.	N.D.	N.D.	N.D.	GU191004	20090951
Mandovi Estuary on the central west coast of India	estuary sediment	15.4669371N	73.7894052E	MES	16.3%	N.D.	N.D.	N.D.	DQ641725	20144707
		15.4669371N	73.7894052E	ZES	0.8%	N.D.	N.D.	N.D.	DQ641729	
Pika site, Southern Mariana Trough	hydrothermal sulfide chimney	12.9536667N	143.6171446E	Pbsc	3.1%	N.D.	N.D.	N.D.	AB293220	20228114
Snail site, Southern Mariana Trough	hydrothermal sulfide mound	12.9191667N	143.6138113E	Fhm5A	2.1%	N.D.	N.D.	N.D.	AB424706	
The Storegga Slide, off Norway	sediment	64.7546667N	5.101448E	CT-9	N.D.	N.D.	N.D.	N.D.	GU055852	20401609
Marmara Sea	deep-sea sediment	40.8383333N	28.0211446E	Ma29	55.0%	N.D.	N.D.	N.D.	HM103846	20668488
Napoli Mud Volcano in the Eastern Mediterranean Sea	hypersaline marine sediment	33.723995N	24.6817863E	NapMat-6	N.D.	N.D.	N.D.	N.D.	HQ443489	21335391
South China Sea	marine sediment	18.9982833N	111.481628E	E505	N.D.	N.D.	N.D.	N.D.	HQ214495	22117845
Hanford Site 300 Area near Richland, USA	subsurface sediments	46.6928N	119.6338W	oxic Ringold	1.5%	N.D.	N.D.	N.D.	HM187505	22122741
CDE hydrothermal field on the Eastern Lau Spreading Center	hydrothermal precipitates	20.6666667S	176.185522W	TVG13	9.2%	N.D.	55.3%	5.1%	GU207318	22443540
Sep reservoir, France	Lake water	46.0166667N	3.014478E	H	2.9%	N.D.	N.D.	N.D.	JF980374	23395876
Guaymas Basin, Gulf of California	cold seep sediment	27.4247167N	111.5034537W	Reference	15.0%	N.D.	N.D.	N.D.	HE774588	23446836
Lake Challa, East Africa	lake water	3.316664S	37.6969733E	-	N.D.	N.D.	N.D.	N.D.	JX990613	23560451
Suiyo Seamount, Izu-Ogasawara arc	sulfide mound	28.5713889N	140.6414224E	Sm4sm	1.8%	N.D.	1.50%	0.0%	AB629531	23626587
Suiyo Seamount, Izu-Ogasawara arc	benthic sand	28.5713889N	140.6414224E	Smhsd	1.3%	N.D.	0.10%	0.0%	AB629591	
Ogasawara Trench	hadopelagic sediment	29.15N	142.814478E	35cmbsf	2.7%	N.D.	10.0%	0.3%	AB583361	23718903
		29.15N	142.814478E	65cmbsf	4.1%	N.D.	10.0%	0.4%		
Lilliput on the southern Mid-Atlantic Ridge	deep-sea vent diffuse fluids	9.5475S	13.2278554W	Lilliput	N.D.	N.D.	N.D.	N.D.	HE579794	23761363
Omakere Ridge,Hikurangi margin	cold seep sediment	40.022S	177.806E	SO191-2-78	1.2%	N.D.	N.D.	N.D.	JN884892	24098632
Archaean site, Southern Mariana Trough	subseafloor massive sulfide deposit	12.9393783N	143.629538E	3AA	81.5%	N.D.	2.50%	2.0%	AB858689	25330135
		12.9393783N	143.629538E	3BA	85.5%	N.D.	6.00%	5.1%	AB858690	
		12.9393783N	143.629538E	3CA	62.5%	N.D.	2.50%	1.6%	AB858691	
		12.9393783N	143.629538E	3DA	78.9%	N.D.	4.90%	3.9%	AB858692	
Brenner Furche in the Central Eastern Alps, Austria	groundwater	47.014N	11.522E	V1	N.D.	N.D.	N.D.	N.D.	FM179185	25536902
Atlantis II Deep BSI, Red Sea	brine seawater	21.346N	38.078E	A-UCL1	8.6%	N.D.	1.20%	0.1%	KJ881842	26192212
Erba Deep BSI, Red Sea		20.730N	38.183E	Ei	1.3%	N.D.	9.90%	0.1%	KJ881914	
Discovery Deep BSI, Red Sea		21.283N	38.053E	Di	1.7%	N.D.	20.9%	0.4%	KJ881987	
		21.346N	38.078E	Ai	5.3%	N.D.	78.1%	4.1%	KJ881994	
Atlantis II Deep BSI, Red Sea		21.346N	38.078E	A-UCL3	90.6%	N.D.	13.5%	12.2%	KJ881996	

		21.346N	38.078E	A-UCL2	48.0%	N.D.	37.4%	18.0%	KJ882000	
South West Indian Ridge	low temperature hydrothermal oxides	37.6587S	50.4656113E	T8	3.8%	N.D.	N.D.	N.D.	JN798475	10.1002/jgrg.20057
		37.7805S	49.6452113E	T13M	3.3%	N.D.	N.D.	N.D.	JN798491	
Kairei hydrothermal field, Central Indian Ridge	deep-sea hydrothermal vent fluid	25.3203233S	70.0375613E	F4-ISCS	12.5%	N.D.	1.30%	0.2%	AB095141	10.1007/s00792-004-0386-3
Eastern Lau Spreading Centre in the Lau Basin, southwest Pacific	deep-sea hydrothermal vent chimney	22.2158S	176.6072W	TVG8AR	N.D.	N.D.	N.D.	N.D.	EU560098	10.1007/s13131-013-0276-6
		20.9280S	176.2401W	TVG11AR	N.D.	N.D.	N.D.	N.D.	EU560099	
Port Klang, Peninsular Malaysia	coastal seawater	3.0005556N	101.3892002E	PK	N.D.	N.D.	N.D.	N.D.	JQ626926	10.1007/s13213-015-1041-1
Mandovi , Goa on the central west coast of India	marine sediment	15.4669371N	73.7894052E	Mandovi	0.6%	N.D.	N.D.	N.D.	JQ257762	10.1016/j.resmic.2010.01.008
Mariner hydrothermal field, Lau basin	hydrothermal chimney	22.3416667S	176.603022W	Snow Chimney	93.5%	N.D.	10.0%	9.4%	AB247816	10.1029/2007JG000636
Kalahari Shield, South Africa	subsurface water	28.25S	26.7811446E	-	N.D.	N.D.	N.D.	N.D.	DQ354739	10.1080/01490450600875696
Evander mine, South Africa	borehole fluid	26.4841805S	29.1124893E	TJA	74.4%	N.D.	N.D.	N.D.	EF446785	10.1080/01490450701572416
Brothers Caldera, Kermadec Volcanic Arc	hydrothermal sulfide chimney	34.862367S	179.058617W	851-3A inside	87.1%	N.D.	N.D.	N.D.	AB247864	10.1080/01490450903304949
		34.862367S	179.058617W	851-3A exterior	100%	N.D.	N.D.	N.D.		
		34.862000S	179.058017E	852-2B inside	96.9%	N.D.	N.D.	N.D.	AB247865	
		34.862000S	179.058017E	852-2B exterior	100%	N.D.	N.D.	N.D.		
Levantine basin, Eastern Mediterranean Sea	cold sepp sediment	32.9357733N	34.780703E	-	N.D.	N.D.	N.D.	N.D.	KF199284	10.1111/1574-6941.12264
Alaminos Canyon 601, Gulf of Mexico	deep sea brine lake	26.3883333N	94.515522W	bottom	3.1%	N.D.	N.D.	N.D.	KP247369	10.1111/gbi.12185
ODP Leg 201 Site 1231, Peru Basin	deep subsurface sediment	12.021075S	81.9062104W	1.8BB	3.7%	N.D.	N.D.	N.D.	AY661828	10.1111/j.1472-4677.2004.00028.x
Lagadas hot springs, Greece	hot spring water	40.575294N	22.8895153E	Lag	N.D.	N.D.	N.D.	N.D.	EF444678	10.3354/ame01333
Hydrate Ridge, Oregon	marine sediment	4.6708333N	125.1071887W	OS_3487	N.D.	N.D.	N.D.	N.D.	KM357114	10.3389/fmars.2014.00044
Ambitle Island, Tutum Bay, Papua New Guinea	sediment	4.0756789S	153.581461E	-	N.D.	N.D.	N.D.	N.D.	GU137351	Akerman, N.H., 2009. Ph.D. Thesis
Zveplenica karst spring, Gorenja Trebusa, Slovenia	gray microbial mat	46.0939472N	13.8367113E	-	N.D.	N.D.	N.D.	N.D.	KT072261	unpublised
Iheya North, Mid-Okinawa Trough	hydrothermal sediment	22.3416667S	176.603022W	-	N.D.	N.D.	N.D.	N.D.	AB197214	unpublised
South Africa	gold mine	28.25S	26.7811446E	-	N.D.	N.D.	N.D.	N.D.	DQ088712	unpublised
ODP Leg 195 Site 1200, South Chamorro Seamount, Mariana arc	crustal fluids	13.7833333N	146.0011446E	-	N.D.	N.D.	N.D.	N.D.	EF414503	unpublised
Hook Ridge crater,Bransfield Strait, Antarctica	marine sediment	62.12S	57.17W	-	N.D.	N.D.	N.D.	N.D.	FM868058	unpublised
Trollosen, Svalbard	thermal spring water	76.70N	16.23E	-	N.D.	N.D.	N.D.	N.D.	JN083980	unpublised
Site U1317 Hole A, Porcupine seabight, Atlantic Ocean	subseafloor sediment	51.38N	11.720522W	-	N.D.	N.D.	N.D.	N.D.	JN229542	unpublised
ODP Site 1226, eastern equatorial Pacific	marine sediment	3.09448N	90.8201637W	-	N.D.	N.D.	N.D.	N.D.	JN675937	unpublised
Orca Basin, Gulf of Mexico	marine sediment	26.938N	91.286W	-	N.D.	N.D.	N.D.	N.D.	KP204816	unpublised
northeastern Gulf of Mexico	marine sediment	29.50N	86.6667W	-	N.D.	N.D.	N.D.	N.D.	KT424616	unpublised
northeastern Gulf of Mexico	marine sediment	29.4167N	87.25W	-	N.D.	N.D.	N.D.	N.D.	KT424711	unpublised
northeastern Gulf of Mexico	marine sediment	29.5792N	86.1583W	-	N.D.	N.D.	N.D.	N.D.	KT424865	unpublised
Aeolian Volcanic Archipelago, Southern Tyrrhenian Sea	filamentous microbial biofilm	38.64N	15.11E	-	N.D.	N.D.	N.D.	N.D.	KX524610	unpublised
Maizuru Bay, Japan	surface sediment	35.4971N	135.3766E	-	N.D.	N.D.	N.D.	N.D.	LC049961	unpublised
Vlasa spring, Bulgaria	hot spring water	42.02N	23.99E	-	N.D.	N.D.	N.D.	N.D.	LT575993	unpublised

Table S4. List of the locations where 16S rRNA genes of Ca. Hydrothermarchaeota were detected (IMNGS)

Location	isolated source	latitude	Longitude	SampleName	#total reads	#MBGE reads	%MBGE reads	Run ID	SRA Study ID	Reference (pubmed ID or DOI)
Site 115, Northern East China Sea	seawater	28.97 N	121.83 E	115A	26538	1	0.004%	DRR028680		
Site 31, Northern East China Sea	seawater	30.46 N	121.21 E	31A	24391	1	0.004%	DRR028729	DRP002509	27538872
Site 58, Northern East China Sea	seawater	30.1 N	121.8 E	58A	26515	1	0.004%	DRR028738		
Skagerrak, North Sea, Sweden	marine sediment	58.4917 N	9.6298 E	SK1012	32706	52	0.16%	DRR057945	DRP003064	26960392
		30.50 N	121.78 E	HZ2	14562	1	0.01%	DRR060138		
		30.40 N	121.35 E	HZ3	33458	2	0.01%	DRR060139		
		30.23 N	121.93 E	HZ4	58300	23	0.04%	DRR060140		
		29.14 N	121.83 E	SM1	56089	33	0.06%	DRR060142		
		29.11 N	121.64 E	SM2	33343	22	0.07%	DRR060143		
		29.06 N	121.74 E	SM3	13794	15	0.11%	DRR060144		
		28.97 N	121.83 E	SM4	20835	29	0.14%	DRR060145		
		28.94 N	121.78 E	SM5	22123	12	0.05%	DRR060146		
		29.42 N	122.34 E	XSE1	61464	35	0.06%	DRR060147		
		29.42 N	122.22 E	XSE2	33656	13	0.04%	DRR060148		
northern Zhejiang, East China Sea	marine sediment	29.35 N	122.25 E	XSE4	61869	10	0.02%	DRR060150	DRP003373	27917954
		29.30 N	122.08 E	XSE5	12955	3	0.02%	DRR060151		
		29.15 N	122.00 E	XSE6	8686	3	0.03%	DRR060152		
		29.13 N	122.22 E	XSE7	34547	2	0.01%	DRR060153		
		29.66 N	122.00 E	XSH1	24327	24	0.10%	DRR060154		
		29.51 N	121.59 E	XSH4	33736	3	0.01%	DRR060157		
		28.91 N	122.25 E	YS1	31084	1	0.003%	DRR060158		
		28.88 N	122.11 E	YS3	16637	1	0.01%	DRR060160		
		29.91 N	122.20 E	ZSE4	15141	1	0.01%	DRR060167		
		29.90 N	122.49 E	ZSE6	34409	4	0.01%	DRR060169		
		29.89 N	122.29 E	ZSE7	50948	8	0.02%	DRR060170		
Lake Nasser, Egypt	fish gut	22.585889 N	32.302472 E	GTKO_S112_L001_R1_001	51044	3	0.01%	DRR119257	DRP003953	unpublished
Snakepit, Mid-Atlantic ridge	iron-rich mat	23.3693611 N	44.9523056 W	J2667_B4	4100	18	0.44%	ERR580000	ERP006666	25760332
IMOS National Reference Stations, Timor Sea	seawater	12.3382 S	130.6952 E	OSD191_2014-07-02_0m_NPL022	2044449	2	0.0001%	ERR867782	ERP009703	10.1186/s13742-015-0066-5
		55.19 N	4.23 W	PMLBenthicSurvey9	77741	1	0.001%	ERR792517		
		55.19 N	4.23 W	PMLBenthicSurvey10	90449	1	0.001%	ERR792518		
		55.19 N	4.23 W	PMLBenthicSurvey24	28873	4	0.01%	ERR792532		
Western English Channel Observatory	marine sediment	55.19 N	4.23 W	PMLBenthicSurvey27	31094	2	0.01%	ERR792535	ERP009928	unpublished
		55.19 N	4.23 W	PMLBenthicSurvey28	17052	1	0.01%	ERR792536		
		55.19 N	4.23 W	PMLBenthicSurvey29	29319	6	0.02%	ERR792537		
		55.19 N	4.23 W	PMLBenthicSurvey30	23039	5	0.02%	ERR792538		
		55.19 N	4.23 W	PMLBenthicSurvey31	26448	2	0.01%	ERR792539		
		9.82S	150.82E	1_1	55049	1	0.002%	ERR1056312		
		9.82S	150.82E	1_1	42185	3	0.01%	ERR1056313		
		9.82S	150.82E	12_1	114062	4	0.004%	ERR1056314		
		9.82S	150.82E	12_1	38828	60	0.15%	ERR1056315		
		9.82S	150.82E	2_1	48577	9	0.02%	ERR1056316		
		9.82S	150.82E	2_1	12193	260	2.13%	ERR1056317		
		9.82S	150.82E	31_1	248004	328	0.13%	ERR1056318		
		9.82S	150.82E	31_1	165775	22305	13.45%	ERR1056319		
		9.82S	150.82E	32_2	26992	2	0.01%	ERR1056321		
		9.82S	150.82E	4_1	26185	6	0.02%	ERR1056323		
Upa Up- asina, Normanby Island, Papua New Guinea	cold seep sediment	9.82S	150.82E	5_1	92404	1	0.001%	ERR1056324	ERP012766	26887661
		9.82S	150.82E	5_1	65335	91	0.14%	ERR1056325		
		9.82S	150.82E	6_3	47014	4	0.01%	ERR1056329		
		9.82S	150.82E	A_1	51893	145	0.28%	ERR1056330		
		9.82S	150.82E	A_1	39220	11096	28.29%	ERR1056331		
		9.82S	150.82E	B_1	53294	26	0.05%	ERR1056332		
		9.82S	150.82E	B_1	51074	1164	2.28%	ERR1056333		
		9.82S	150.82E	G_1	111074	1	0.00%	ERR1056334		
		9.82S	150.82E	G_1	54284	46	0.08%	ERR1056335		
		9.82S	150.82E	Sa_1	91403	110	0.12%	ERR1056336		
		9.82S	150.82E	Sa_1	69339	2905	4.19%	ERR1056337		
		28.6330555 N	92.0269444 W	Sta A 0-1 cm 1	44288	2	0.005%	ERR2233280		
Gulf of Mexico	marine sediment	28.6330555 N	92.0269444 W	Sta A 0-1 cm 2	43364	3	0.01%	ERR2233281	ERP016794	29343830
		28.6330555 N	92.0269444 W	Sta A 2-3 cm 2	51139	1	0.002%	ERR2233283		

		29.0111111 N	91.9002777 W	Sta 3 12-14 cm 2	54853	1	0.002%	ERR2233295		
Red Sea aquaculture	water	21.8060701 N	39.0352522 E	S1A_1	791419	3	0.0004%	ERR1597357	ERP016950	10.1016/j.aquaculture.2017.08.014
Faroe Shetland Channel	deep-sea sediment	61.5833 N	4.2500 W	13	67828	3	0.004%	ERR1685359	ERP017791	28533547
		61.1331667 N	2.173 W	70	39605	2	0.01%	ERR1685416		
Cala Montgó, Spain	seawater	42.1146111 N	3.1672222 E	Sample7	2641097	19	0.0007%	ERR2073740	ERP024352	29326681
Mediterranean Sea	seawater	42.487 N	3.165 E	BZZ_DU_TA	1083062	1	0.0001%	ERR2196991	ERP090011	EMOSE (2017)
Tekslo, Norway	kelp material	60.161767N	5.039517E	Kelp biofilm	7073	1	0.01%	SRR071724	SRP004175	22763650
Arctic mid-ocean ridge	marine sediment	73.3565 N	7.565 E	GC6-87	3798	32	0.84%	SRR396772	SRP009131	23027979
		73.3565 N	7.565 E	GC6-95	3863	4	0.10%	SRR396773		
Terrebonne Bay, the northern Gulf of Mexico	marine sediment	28.875 N	90.428 W	H3031: OMSA31	37500	2	0.01%	SRR1631172	SRP015731	25939270
southwestern Taiwan	cold seep sediment	22.242644 N	119.9038011 E	MD-178-3280-150cmbsf	12990	1	0.01%	SRR978812	SRP029906	10.1016/j.jseaes.2014.02.014
Atlantis II, Red Sea	brine water	21.60528 N	38.2025 E	ATII-I	7023	11	0.16%	SRR1016448	SRP029993	25295031
Bohai Sea		38.3375833 N	120.195 E	B41-A	6863	60	0.87%	SRR1010961		
		38.3327833 N	119.7851167 E	B42-A	7044	45	0.64%	SRR1010962		
		39.18395 N	120.0638333 E	B51-A	5586	9	0.16%	SRR1010963		
North Yellow Sea	marine sediment	38.13535 N	123.2479 E	B13-A	5113	3	0.06%	SRR1010966	SRP030955	25501892
South Yellow Sea		36.9883333 N	123.4243167 E	B08-A	5367	5	0.09%	SRR1010967		
		35.0128 N	122.3349833 E	H13-A	5028	31	0.62%	SRR1010969		
norh East China Sea		31.2725833 N	125.3316667 E	E05-A	5646	1	0.02%	SRR1010972		
Arctic Ocean	marine sediment	53.9822333 N	170.7143 E	Arc-BL03	6607	1	0.02%	SRR1063376	SRP034720	10.1007/s13131-017-1030-2
Pearl Estuary, China	marine sediment	22.25955 N	113.8156 E	A08M-A	5716	9	0.16%	SRR1138532	SRP035578	25467555
		22.07071 N	113.9002 E	F412S-A	5970	88	1.47%	SRR1138534		
		22.07071 N	113.9002 E	F412M-A	6478	6	0.09%	SRR1138535		
		22.19006 N	113.7687 E	C2S-A	5643	9	0.16%	SRR1138537		
		22.16266 N	113.7131 E	C3S-A	6380	22	0.34%	SRR1138538		
		22.00073 N	113.9686 E	F414S-A	5262	2	0.04%	SRR1138539		
Shangyu Industrial Area, Hangzhou Bay	sediment	30.1427N	120.8904E	S4-Feb	4061	222	5.47%	SRR1187254	SRP039368	26960319
		30.1427N	120.8904E	S5-Feb	4036	139	3.44%	SRR1187258		
		30.1427N	120.8904E	S6-Feb	4325	181	4.18%	SRR1187262		
		30.1427N	120.8904E	S2-May	9596	111	1.16%	SRR1187266		
		30.1427N	120.8904E	S4-May	9384	29	0.31%	SRR1187270		
		30.1427N	120.8904E	S2-Aug	20182	2068	10.25%	SRR1187274		
		30.1427N	120.8904E	S4-Aug	22797	2202	9.66%	SRR1187278		
		30.1427N	120.8904E	S2-Nov	7158	28	0.39%	SRR1187282		
		30.1427N	120.8904E	S4-Nov	4786	13	0.27%	SRR1187286		
	water	30.1427N	120.8904E	W4-Feb	3963	29	0.73%	SRR1187290		
		30.1427N	120.8904E	W5-Feb	3558	7	0.20%	SRR1187292		
Thuwal Seeps, Red Sea	cold seep fluid	22.3021N	39.0279E	red sea microbial 16S	116318	178	0.15%	SRR1491768	SRP040612	26059861
Sundarbans, India	marine sediment	21.628010 N	88.563267 E	Archaea_Sundarbans_WB	24884	187	0.75%	SRR1632258	SRP041568	26981367
		21.628010 N	88.563267 E	Archaea_Sundarbans_WB	27768	89	0.32%	SRR1632259		
		21.628010 N	88.563267 E	Archaea_Sundarbans_WB	28442	20	0.07%	SRR1632260		
		21.628010 N	88.563267 E	Archaea_Sundarbans_WB	32491	80	0.25%	SRR1632261		
		21.628010 N	88.563267 E	Archaea_Sundarbans_WB	33999	329	0.97%	SRR1632262		
		21.628010 N	88.563267 E	Archaea_Sundarbans_WB	21242	57	0.27%	SRR1632263		
Atlantis II Deep, Red Sea	hot brine sediment	21.3455333 N	38.0839833 E	Atlantis II Deep - Section 1	1640	275	16.77%	SRR1351539	SRP042352	22916172
Discovery Deep, Red Sea		21.28475 N	38.0484E	Discovery Deep - Section 1	20916	23	0.11%	SRR1351663		
Valparaiso, Chile	marine sediment	33.01535 S	71.6222167 W	VAG_014	15403	24	0.16%	SRR1346080	SRP043010	unpublished
Valparaiso, Chile		33.02475 S	71.6220833 W	VAG_015	12482	7	0.06%	SRR1346081		
Southwest Indian Ridge	marine sediment	37.8333 S	49.6167 E	M8	7503	6	0.08%	SRR1573328	SRP046759	27621725
off Manzanillo, Mexico	oxygen minimum zone seawater	18.9 N	104.5 W	85m_0.2-1.6micron_Station6	18761	12	0.06%	SRR1824243	SRP052876	26441925
		18.9 N	104.5 W	100m_0.2-1.6micron_Station6	17299	41	0.24%	SRR1824244		
		18.9 N	104.5 W	125m_0.2-1.6micron_Station6	18126	58	0.32%	SRR1824246		
		18.9 N	104.5 W	300m_0.2-1.6micron_Station6	19314	22	0.11%	SRR1824247		
		18.9 N	104.5 W	100m_1.6-30micron_Station6	19034	2	0.01%	SRR1824251		
		18.9 N	104.5 W	100m_>30micron_Station6	15149	9	0.06%	SRR1824256		
		18.9 N	104.5 W	300m_>30micron_Station6	6660	6	0.09%	SRR1824259		
East China Sea	marine sediment	27.79 N	126.89 E	S1	34111	89	0.26%	SRR1801945	SRP054961	unpublished
northern Gulf of Mexico	hypoxia seawater	28.91 N	92.38 W	H5	54383	3	0.01%	SRR1952965	SRP056891	25818237
		28.66 N	92.38 W	H7	79246	2	0.003%	SRR1952967		
		18.20 N	104.23 W	Exp1_0.1L_r1_sv	3744	6	0.16%	SRR2026399		
		18.20 N	104.23 W	Exp1_0.1L_r2_sv	87498	50	0.06%	SRR2026400		
		18.20 N	104.23 W	Exp1_0.1L_r3_sv	29408	23	0.08%	SRR2026401		
		18.20 N	104.23 W	Exp1_0.1L_r4_sv	13521	11	0.08%	SRR2026403		

off Manzanillo, Mexico	oxygen minimum zone seawater	18.20 N	104.23 W	Exp1_0.1L_r5_sv	37774	36	0.10%	SRR2026405	SRP058343	26082766
		18.20 N	104.23 W	Exp1_1.0L_r1_sv	11567	2	0.02%	SRR2026407		
		18.20 N	104.23 W	Exp1_1.0L_r2_sv	4201	2	0.05%	SRR2026409		
		18.20 N	104.23 W	Exp1_1.0L_r3_sv	11066	15	0.14%	SRR2026411		
		18.20 N	104.23 W	Exp1_1.0L_r5_sv	4940	1	0.02%	SRR2026414		
		18.20 N	104.23 W	Exp1_5.0L_r1_sv	5633	1	0.02%	SRR2026417		
		18.20 N	104.23 W	Exp1_5.0L_r2_sv	4896	4	0.08%	SRR2026421		
		18.20 N	104.23 W	Exp1_5.0L_r3_sv	12886	15	0.12%	SRR2026423		
		18.20 N	104.23 W	Exp1_5.0L_r4_sv	11782	12	0.10%	SRR2026425		
		18.20 N	104.23 W	Exp1_5.0L_r5_sv	9290	11	0.12%	SRR2026428		
Paranagua, Brazil	marine sediment	25.54880 S	48.47157 W	PAR_32	17680	2	0.01%	SRR2032972	SRP058359	unpublished
		25.54879 S	48.47172 W	PAR_51	32617	5	0.02%	SRR2032977		
		25.54879 S	48.47174 W	PAR_53	116798	7	0.01%	SRR2032979		
Sichuan pixian, China	doubanjiang-meju	30.48 N	103.53 E	doubanjiang-meju	506331	7	0.00%	SRR2086466	SRP060307	10.1016/j.foodchem.2016.09.104
LTER site Helgoland, North Sea	Subsurface seawater	54.174181 N	7.884658 E	SWWP	392722	1	0.0003%	SRR2103876	SRP061219	unpublished
		54.174181 N	7.884658 E	SWUP	255512	1	0.0004%	SRR2103935		
		54.174181 N	7.884658 E	SNHH	409058	1	0.0002%	SRR2103966		
LTER site Helgoland, North Sea	Subsurface seawater	54.174181 N	7.884658 E	SWZP	179879	1	0.001%	SRR2104095	SRP061220	unpublished
		54.174181 N	7.884658 E	SXUY	239435	1	0.0004%	SRR2104113		
Wanning City, Hainan Province, China	soil	18.733 N	110.200 E	BR1	40749	1	0.002%	SRR2226501	SRP062990	26903995
Southern Atlantic Ridge	hydrothermal sediment	13.5933333S	14.51W	SMAR-TA	14595	11473	78.61%	SRR2580814	SRP064585	10.1002/2017jg003852
Tuscan Island Elba, Italy	cold seep sediment	42.74372 N	10.11852 E	Elba Pomonte reference site 1	18450	5	0.03%	SRR2646293	SRP064784	27065954
		42.74372 N	10.11852 E	Elba Pomonte reference site 2	11253	273	2.43%	SRR2646295		
		42.74372 N	10.11852 E	Elba Pomonte reference site 3	13078	173	1.32%	SRR2646304		
Yangtze River, China	freshwater	31.77 N	120.96 E	24XLJ_SSB1	137109	1	0.0007%	SRR3944731	SRP079372	29351813
		31.77 N	120.96 E	24XLJ_SSB4	102320	1	0.001%	SRR3944732		
		31.77 N	120.96 E	24XLJ_SSL	67857	97	0.14%	SRR3944733		
		31.77 N	120.96 E	24XLJ_SSR	58118	1	0.002%	SRR3944734		
		28.8 N	104.42 E	25GC_SSB	142792	3	0.002%	SRR3944741		
		31.46 N	118.34 E	20WHU_FSL	65613	3	0.005%	SRR3944977		
		32.18 N	119.66 E	23ZJ_FSL	91383	36	0.04%	SRR3944998		
